# The miR-1224-5p/TNS4/EGFR axis inhibits tumour progression in oesophageal squamous cell carcinoma

**DOI:** 10.1038/s41419-020-02801-6

**Published:** 2020-07-30

**Authors:** Zhi-Zhou Shi, Wen-Jun Wang, Yun-Xia Chen, Ze-Wen Fan, Xiu-Feng Xie, Li-Yan Yang, Chen Chang, Yan Cai, Jia-Jie Hao, Ming-Rong Wang, Jie Bai

**Affiliations:** 1 Medical School, Kunming University of Science and Technology, Kunming, 650500 China; 2 State Key Laboratory of Molecular Oncology, National Cancer Center/National Clinical Research Center for Cancer/Cancer Hospital, Chinese Academy of Medical Sciences and Peking Union Medical College, Beijing, 100021 China

**Keywords:** Oesophageal cancer, Mechanisms of disease

## Abstract

Oesophageal squamous cell carcinoma (ESCC) is a common and aggressive malignancy. Although many molecular alterations have been observed in ESCC, the mechanisms underlying the development and progression of this disease remain unclear. In the present study, miR-1224-5p was identified to be downregulated in ESCC tissues compared to normal tissues, and its low expression was correlated with shorter survival time in patients. In vitro experiments showed that miR-1224-5p inhibited the proliferation, colony formation, migration and invasion of ESCC cells. Mechanistic investigation revealed that miR-1224-5p directly targeted TNS4 and inhibited its expression, which led to the inactivation of EGFR-EFNA1/EPHA2-VEGFA (vascular endothelial growth factor A) signalling. Experiments in vivo confirmed the suppressive effect of miR-1224-5p on oesophageal cancer cells. By immunohistochemistry analysis of ESCC specimens, we found that TNS4 expression was positively correlated with that of VEGFA, and was significantly associated with lymph node metastasis and shorter survival time in patients. Together, our data suggest that miR-1224-5p downregulation is a frequent alteration in ESCC that promotes cell proliferation, migration, invasion and tumour growth by activating the EGFR-EFNA1/EPHA2-VEGFA signalling pathway via inhibition of TNS4 expression. Decreased miR-1224-5p and elevated TNS4 are unfavourable prognostic factors for ESCC patients.

## Introduction

Oesophageal squamous cell carcinoma (ESCC) is one of the most common and aggressive malignancies worldwide, and is the fourth leading cause of cancer-related death in China^1,2^. Although early diagnosis and cancer treatment options have been developed, the 5-year survival of ESCC is still poor^3^. Therefore, it is very important and urgent to study the molecular mechanisms of oesophageal cancer progression, and develop new diagnostic and prognostic methods in ESCC.

MicroRNAs (miRNAs) are small non-coding RNAs that have ~22 nucleotides^4^. miRNAs play critical roles in cancer development by binding to the 3′-UTR of target genes and downregulating their expression. Several miRNAs have been reported to be involved in oesophageal cancer progression. miR-204-5p regulates cell proliferation and invasion by directly targeting interleukin-11 in ESCC^5^. miR-134 inhibits tumour growth and lymph node metastasis by targeting PLXNA1 and blocking the downstream MAPK signalling pathway^6^. miR-29c expression is decreased in tumours and serum of ESCC patients, and its overexpression abolishes 5-FU chemoresistance both in vitro and in vivo by targeting FBXO31 (ref. ^7^). Downregulation of miR-145 has been detected, and miR-145 directly targets and regulates PLCE1 to suppress cell proliferation and migration in ESCC^8^. However, the roles and mechanisms of many other miRNAs in ESCC are still largely unknown.

In the present study, we screened the differentially expressed miRNAs in ESCC using microarray and found that miR-1224-5p was downregulated, and its low expression was correlated with shorter survival time of patients. Although previous studies reported that miR-1224-5p suppressed tumour metastasis by targeting FAK in intestinal-type gastric cancer, and inhibited the proliferation and invasion of glioma cells by targeting CREB1 (refs. ^6,9^), the roles and underlying mechanisms of miR-1224-5p in oesophageal cancer and other types of cancer are still largely unclear. Therefore, we explored the detailed mechanism underlying miR-1224-5p loss in ESCC.

## Materials and methods

### Patients and tissue specimens

Fresh tumour tissues from 227 ESCC patients were obtained from surgical resection specimens collected by the Department of Pathology at the Cancer Hospital, Chinese Academy of Medical Sciences and Peking Union Medical College (CAMS and PUMC), Beijing, China. All patients signed informed consent forms and received no treatment before surgery. Morphological normal operative margin tissues and primary tumour tissues were separated by experienced pathologists, and immediately stored at −80 °C. This study was approved by the Ethics Committee of Cancer Institute (Hospital), CAMS and PUMC.

### Cell culture and treatments

The ESCC cell lines KYSE30, KYSE140, KYSE150, KYSE180, KYSE450 and KYSE510 were generously provided by Dr. Y. Shimada (Kyoto University, Kyoto, Japan). EC109 cell line was purchased from the cell bank of Institute of Basic Medical Sciences, Chinese Academy of Medical Sciences. TE10 cell line was purchased from the Shanghai Institute of Cell Biology, Chinese Academy of Sciences. The human oesophageal epithelial cell line Het1A was purchased from ATCC. The cells were cultured in RPMI-1640 medium with 10% foetal bovine serum (FBS, Gibco, Carlsbad, CA, USA), penicillin (100 U/mL) and streptomycin (100 µg/mL) at 37 °C with 5% CO_2_. All of the above cell lines were authenticated by short tandem repeat profiling and were routinely tested for mycoplasma contamination.

The miR-1224-5p inhibitor, miR-1224-5p mimic, TNS4 siRNAs, EGFR siRNA and negative control sequences were designed, and synthesised by GenePharma Co., Ltd (Shanghai, China). The sequences are as follows: miR-1224-5p inhibitor, 5′-CCACCUCCCGAGUCCUCAC-3′; miRNA inhibitor NC, 5′-CAGUACUUUUGUGUAGUACAA-3′; miR-1224-5p mimic sense, 5′-GUGAGGACUCGGGAGGUGG-3′, antisense, 5′-ACCUCCCGAGUCCUCACUU-3′; TNS4 si1 sense, 5′-GCAUCUCAAUCCCUUGCAUTT-3′, antisense, 5′-AUGCAAGGGAUUGAGAUGCTT-3′; TNS4 si2 sense, 5′-CCAAAGGAGUGCAUCUCAATT-3′, antisense, 5′-UUGAGAUGCACUCCUUUGGTT-3′; siEGFR sense, 5′-CUCUGGAGGAAAAGAAAGU-3′, antisense, 5′-ACUUUCUUUUCCUCCAGAG-3′; and negative control sense, 5′- UUCUCCGAACGUGUCACGUTT-3′, antisense, 5′-ACGUGACACGUUCGGAGAATT-3′.

The pGCMV/EGFP/miR-1224-5p vector was obtained from GenePharma Co., Ltd. Stable cell lines were selected by using 10 μg/mL blasticidin for 4 weeks.

Transfection and co-transfection were performed using Lipofectamine 3000 (Thermo Scientific, Carlsbad, CA, USA) according to the manufacturer’s instructions.

### Total RNA extraction and quantitative real-time PCR

RNA extraction and quantitative real-time PCR (qRT-PCR) analysis were carried out as described previously^10^. The primers are shown in Supplementary Table 1. GAPDH/U6 was used as an internal control.

### miRNA and mRNA microarray

Agilent human gene expression and miRNA microarrays were performed as described previously^11^. The data were extracted and analysed by Agilent Feature Extraction Software. The raw data were normalised using quantile normalisation and consequentially analysed by GeneSpring GX software (Agilent Technologies).

### Western blotting analysis

Western blotting analysis was carried out as described previously^10^. The following antibodies were used: anti-TNS4 antibody (Abcam, ab192247), anti-VEGFA antibody (Abcam, ab52917), anti-EGFR antibody (Abcam, ab52894), anti-EFNA1 antibody (Abcam, ab124911), anti-p-EPHA2 (Cell Signaling Technology, 6347), anti-EPHA2 antibody (Cell Signaling Technology, 6997) and anti-β-actin (Proteintech, 66009-1-IG).

### Immunoprecipitation analysis

The co-immunoprecipitation (co-IP) method was applied to analyse the interaction between TNS4 and EGFR in KYSE150 and KYSE510 cells, as previously described^12^. After cell lysis, 50 μl of the protein G-agarose suspension was mixed with the sample and incubated for 4 h at 4 °C. After removing the beads, 5 mg of the anti-EGFR antibody (Abcam, ab52894) was added to the supernatant and then incubated overnight at 4 °C. Protein G-agarose was added to the mixture and incubated overnight at 4 °C. The immunoprecipitates were collected and added to loading buffer. After boiling, the samples were subjected to western blotting assay.

### Dual-luciferase reporter assay

A dual-luciferase reporter assay system (Promega, Madison, WI, USA) was used to detect the binding between miR-1224-5p and the 3′-UTR of TNS4. After co-transfection with luciferase reporter vector and miR-1224-5p mimic for 48 h, the luciferase activity was measured.

### Cell proliferation and colony formation assays

Cell proliferation was detected by using Cell Counting Kit-8 (CCK-8, Dojindo Laboratories, Kumamoto, Japan), as described previously^10^. In the colony formation assay, 10^3^ cells were seeded into six-well plates and cultivated for 8–10 days in RPMI-1640 with 10% FBS. After removing the medium, cells were washed using PBS and stained using crystal violet (Sigma-Aldrich, MO, USA) for 30 min at room temperature. Colonies were visualised and quantitated.

### Transwell assay

Transwell assays were carried out as described previously^10^.

### Immunohistochemistry analysis

Immunohistochemical analysis was performed as described previously^12^. Samples were incubated with anti-TNS4 antibody (Abcam, ab82178) and anti-VEGFA antibody (Abcam, ab52917) overnight at 4 °C followed by treatment with secondary antibody (Santa Cruz). The signals were visualised using PV-9000 and DAB kits (ZhongShan, China). The staining results were defined as positive expression and negative expression.

### Enzyme-linked immunosorbent assay

The vascular endothelial growth factor A (VEGFA) concentrations in the culture media were examined by using a human VEGFA enzyme-linked immunosorbent assay (ELISA) kit (ABclonal, USA). The measurements were performed according to the manufacturer’s instructions and acquired with a microplate reader (Molecular Devices, USA) at 450 nm.

### Xenograft assays in nude mice

Animal studies were performed in accordance with the protocols approved by the Animal Ethics Committee of Kunming University of Science and Technology. Sixteen, 6–8 weeks, female BALB/c mice were randomly divided into four groups and subcutaneously injected with 2 × 10^6^ cells for each of the stably transfected KYSE150 and KYSE510 cells. Tumour size was measured every 7 days for a total of 21 days. Mice were euthanized at the end of experiments. Then, tumours were dissected, weighed and analysed. Tumour sizes were calculated by the formula: width × length × height/2. Immunohistochemistry (IHC) was performed on 5-μm sections of paraffin-embedded subcutaneous tumours to detect the protein expression of TNS4 and VEGFA. qRT-PCR was applied to the RNA of subcutaneous tumours to evaluate the expression of miR-1224-5p, TNS4, EFNA1 and VEGFA.

### Statistical analysis

Statistical analyses were performed using GraphPad Prism 6 (La Jolla, CA, USA). All quantitative data are presented as the mean ± SD. Student’s *t*-test, *χ*^2^ test and ANOVA were used to evaluate the data. Overall survival was estimated by the Kaplan–Meier method with the log-rank test. *P* < 0.05 was considered statistically significant.

## Results

### Screening for ESCC-associated miRNAs

**Fig. 1:**
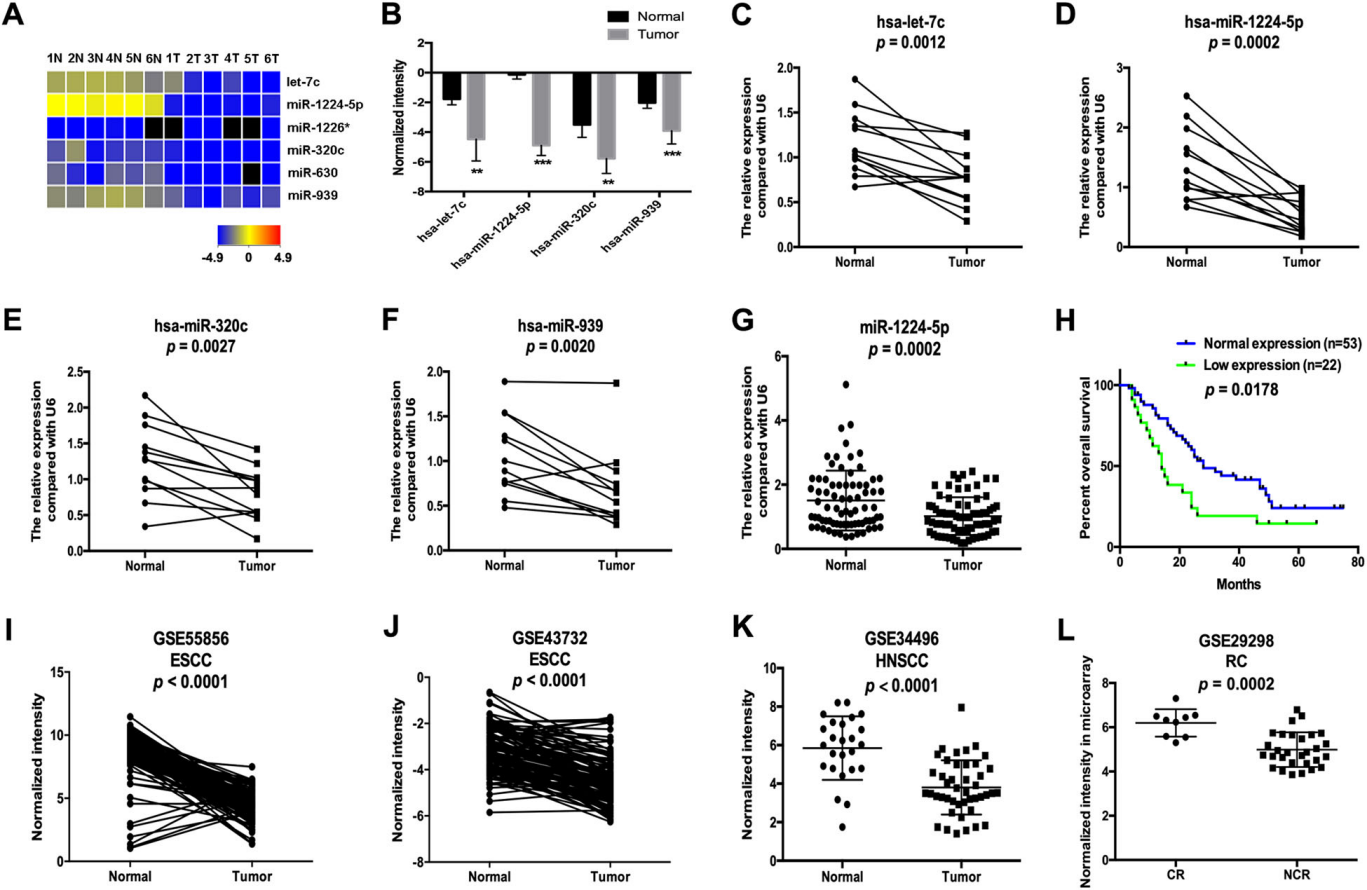
Microarray screening of ESCC-associated miRNAs. **a** Heatmap showing the differentially expressed miRNAs in six ESCC tissues and adjacent non-malignant tissues. **b** The normalised intensities of hsa-let-7c, hsa-miR-1224-5p, hsa-miR-320c and hsa-miR-939 in the microarray assay. **c**–**f** qRT-PCR was used to analyse the expression of hsa-let-7c, hsa-miR-1224-5p, hsa-miR-320c and hsa-miR-939 in ESCC tissues (*n* = 12). **g** The expression of miR-1224-5p was examined by qRT-PCR in independent ESCC samples (*n* = 75). **h** Kaplan–Meier curve showing a positive correlation between low miR-1224-5p expression and lower survival rates in ESCC samples (*n* = 75). **i**–**l** Transcript levels of miR-1224-5p in normal tissues and ESCC tissues from GSE55856 and GSE43732, in normal tissues and HNSCC tissues from GSE34496, and in tissues from locally advanced rectal cancer patients with incomplete (NCR) and complete responses (CR) to neoadjuvant chemoradiotherapy (capecitabine + oxaliplatin + 45 Gy of pelvic conformal radiotherapy) from GSE29298; ** *p* < 0.01, *** *p* < 0.001.

We performed a miRNA microarray in six ESCC tissues and adjacent non-malignant tissues to identify ESCC-associated miRNAs, and six miRNAs, including let-7c, miR-1224-5p, miR-1226*, miR-320c, miR-630 and miR-939, were differentially expressed with thresholds of *p* < 0.05 and fold change > 2.0 (Fig. 1a, b). The decrease in let-7c, miR-1224-5p, miR-320c and miR-939 in ESCC tissues was verified in an independent set of ESCC samples using qRT-PCR (*n* = 12, Fig. 1c–f). Because the expression pattern and functional role of miR-1224-5p in cancer, especially ESCC, are largely unknown, we selected it for further study. In another 75 ESCC samples, reduction of miR-1224-5p was confirmed by qRT-PCR, and its low expression was significantly associated with lymph node metastasis, advanced stage and shorter survival time of patients (Fig. 1g, h, Table 1). Downregulation of miR-1224-5p was also confirmed by analysing the ESCC datasets (GSE55856 and GSE43732, Fig. 1i, j). miR-1224-5p was also decreased in head and neck squamous cell carcinoma (HNSCC), and its low expression was positively correlated with an incomplete response to neoadjuvant chemoradiotherapy (capecitabine + oxaliplatin + 45 Gy of pelvic conformal radiotherapy) in locally advanced rectal cancer patients (Fig. 1k, l) by analysing datasets of GSE34496 and GSE29298. Additionally, its low expression was also associated with asbestos exposure in non-small cell lung cancer (NSCLC, Supplementary Fig. 1) in the GSE25508 dataset.

**Table 1:** The correlation between miR-1224-5p expression and clinicopathological parameters.

Parameter	miR-1224-5p		
	Low expression	Normal expression	*p-*Value
Gender			0.0679
Male	14	44	
Female	8	9	
Age			0.3889
≤60	8	14	
>60	14	39	
pT			0.1231
T1–2	5	5	
T3–4	17	48	
pN			0.009
N0	6	32	
N1	16	21	
Stage			0.035
I–II	5	26	
III–IV	17	27	
Grade			0.3071
1–2	15	42	
3	7	11	

### Gain of miR-1224-5p inhibits and loss of miR-1224-5p promotes the proliferation, colony formation, migration and invasion of oesophageal cancer cells

**Fig. 2:**
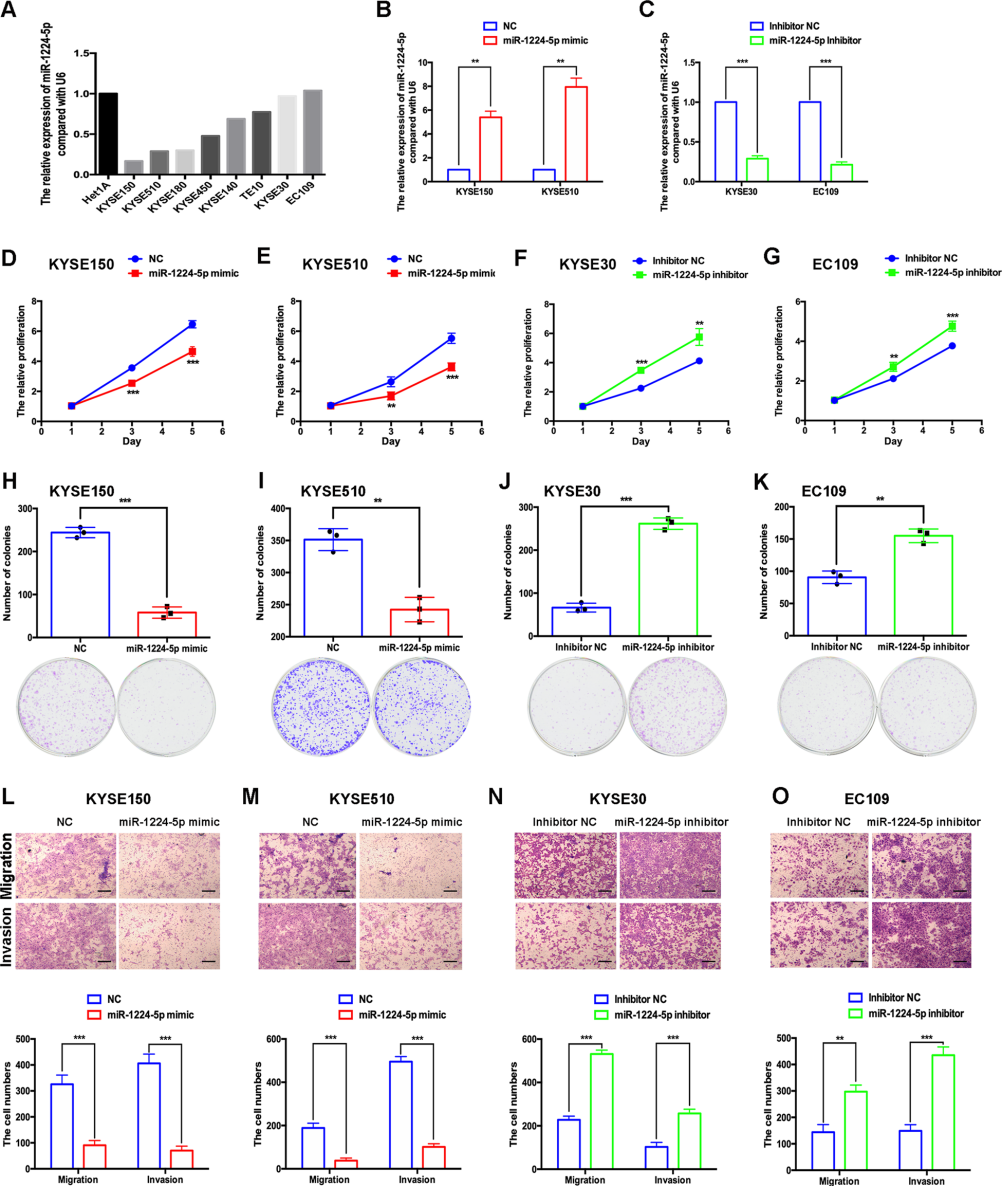
Gain of miR-1224-5p inhibits and loss of miR-1224-5p promotes the proliferation, colony formation, migration and invasion of oesophageal cancer cells. **a** The expression of miR-1224-5p in ESCC cell lines and the human oesophageal epithelial cell line Het1A was examined by qRT-PCR. **b**, **c** The transfection efficiencies of mimic and inhibitor of miR-1224-5p in ESCC cell lines were measured by qRT-PCR. **d**–**k** CCK-8 and colony formation assays were used to analyse the proliferation ability of transfected cells. **l**–**o** The migration and invasion abilities of transfected cells were measured by transwell assay. Scale bar, 200 μm. Data are represented as the mean ± SD, and experiments were performed in triplicate; ** *p* < 0.01, *** *p* < 0.001.

To explore the functional role of miR-1224-5p in ESCC, we first detected the expression of miR-1224-5p in ESCC cell lines and human oesophageal epithelial cells (Het1A). miR-1224-5p was expressed in KYSE30 and EC109 cells at high levels, and in KYSE150 and KYSE510 cells at low levels (Fig. 2a). We overexpressed miR-1224-5p in KYSE150 and KYSE510 cells, and silenced it in KYSE30 and EC109 cells by transfecting mimic or inhibitor, respectively (Fig. 2b, c). Overexpression of miR-1224-5p significantly inhibited the proliferation and colony formation of KYSE150 and KYSE510 cells, and knockdown of miR-1224-5p had the opposite effects in KYSE30 and EC109 cells (Fig. 2d–k). We also evaluated the effects of miR-1224-5p on the migration and invasion of ESCC cells. The results showed that overexpression of miR-1224-5p suppressed the migration and invasion of KYSE150 and KYSE510 cells, and silencing miR-1224-5p promoted the migrative and invasive abilities of KYSE30 and EC109 cells (Fig. 2l–o).

### miR-1224-5p directly targets TNS4 in ESCC

**Fig. 3:**
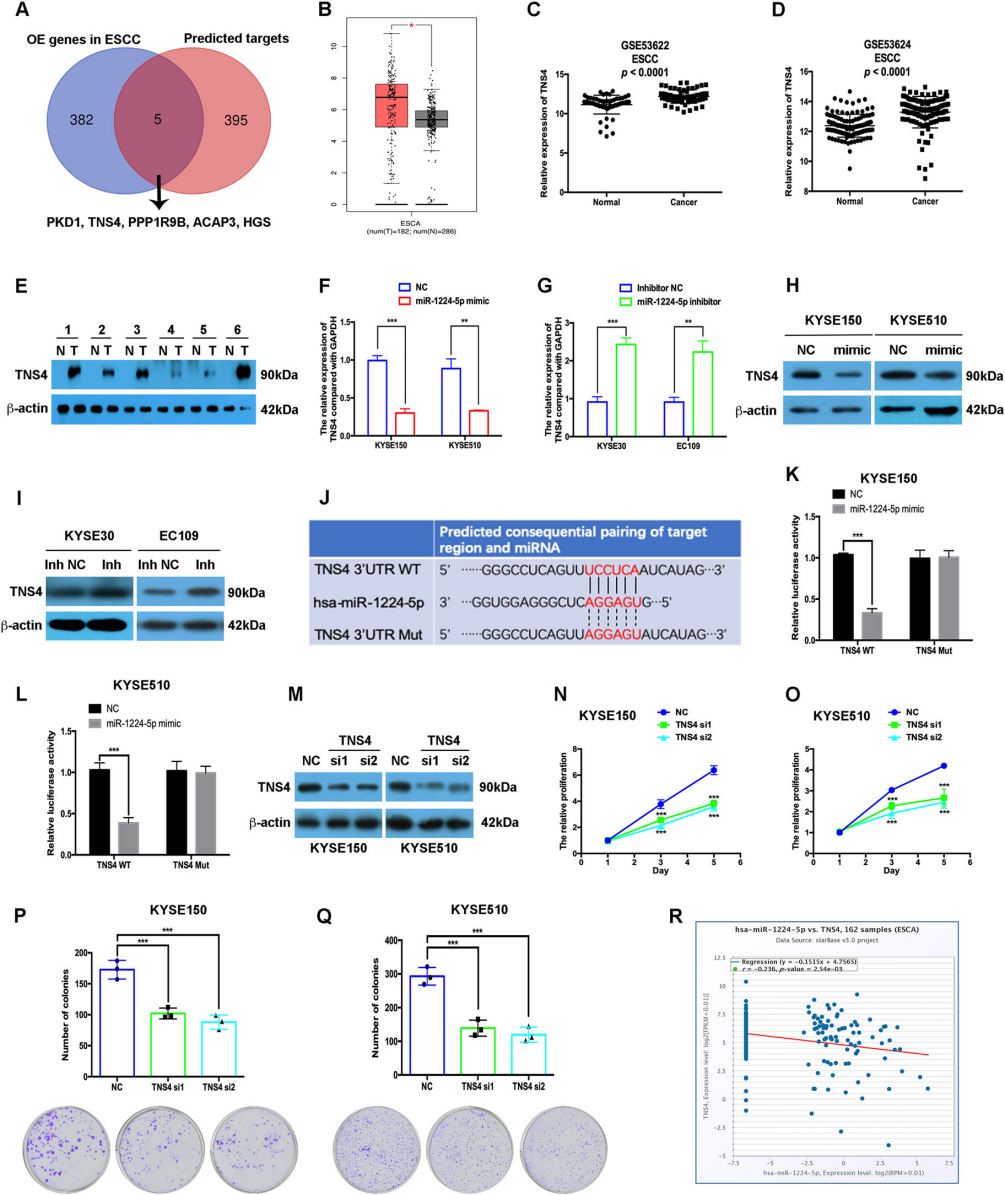
miR-1224-5p directly targets TNS4 in ESCC. **a** The intersection of overexpressed genes in ESCC assayed by microarray and candidate targets predicted by the miRDB database. **b** TNS4 is overexpressed in ESCA by analysing the TCGA dataset using the GEPIA database. **c**, **d** Transcript levels of TNS4 in normal tissues and ESCC tissues from GSE53622 and GSE53624. **e** Western blotting was used to examine TNS4 protein expression in paired ESCC tissues and adjacent non-malignant tissues. **f**–**i** qRT-PCR and western blotting assays were used to measure the mRNA, and protein levels of TNS4. **j** The predicted binding site in the 3′-UTR of TNS4 targeted by miR-1224-5p. **k**, **l** Dual-luciferase activity assay showing the binding affinity between miR-1224-5p and the 3′-UTR of TNS4. **m** The transfection efficiencies of TNS4 siRNAs were detected by western blotting assay. **n**–**q** CCK-8 and colony formation assays were used to analyse the proliferation ability of transfected cells. **r** The negative correlation between miR-1224-5p and TNS4 was identified by analysing the TCGA dataset using the Starbase database. Data are represented as the mean ± SD, and experiments were performed in triplicate; * *p* < 0.05, ** *p* < 0.01, *** *p* < 0.001.

Considering that miR-1224-5p is downregulated in ESCC, its targets are probably upregulated. Therefore, we analysed the intersection of overexpressed genes in ESCC and candidate targets predicted by the miRDB database. Microarray technology was applied to analyse the differentially expressed genes in ESCC by using the same samples as the miRNA microarray assay. Four hundred genes were predicted to be targeted by miR-1224-5p using the miRDB database (Supplementary Table 2), and 387 genes were upregulated in ESCC in microarray data with thresholds of *p* < 0.05 and fold change > 2.0 (Supplementary Tables 3–5). Five genes, including PKD1, TNS4, PPP1R9B, ACAP3 and HGS, were identified in both groups (Fig. 3a). In the TCGA data analysis using the GEPIA database, only TNS4 was identified to be significantly upregulated in oesophageal carcinoma (ESCA, Fig. 3b, Supplementary Fig. 2). ESCC datasets (GSE53622 and GSE53624) further confirmed the overexpression of TNS4 in ESCC (Fig. 3c, d). Figure 3e shows that the protein level of TNS4 in ESCC tissues was higher than that in adjacent non-malignant tissues.

Overexpression of miR-1224-5p decreased the mRNA and protein levels of TNS4 in KYSE150 and KYSE510 cells, and its downregulation had the opposite effects on TNS4 expression in KYSE30 and EC109 cells (Fig. 3f–i). The miRDB database predicted the binding site of miR-1224-5p in the 3′-UTR of TNS4, and a luciferase reporter assay was applied to confirm the binding of miR-1224-5p to the 3′-UTR of TNS4 (Fig. 3j). The miR-1224-5p mimic decreased the relative luciferase activity of the wild-type construct. In contrast, the miR-1224-5p mimic did not affect the relative luciferase activity of the mutant construct in either KYSE150 or KYSE510 cells (Fig. 3k, l).

We further determined the effects of silencing TNS4 on tumorigenic phenotypes in vitro. Similar to the effects of miR-1224-5p overexpression, knockdown of TNS4 also significantly inhibited the proliferation, colony formation, migration and invasion of KYSE150 and KYSE510 cells (Fig. 3m–q, Supplementary Fig. 3). Moreover, the expression of miR-1224-5p was negatively associated with TNS4 expression in oesophageal carcinoma in the Starbase database analysis^13^ (Fig. 3r). The results in Fig. 3 indicate that TNS4 is the direct target of miR-1224-5p.

### miR-1224-5p regulates the autophagic degradation of EGFR, and downstream EFNA1/EPHA2 and VEGFA signalling pathways

**Fig. 4:**
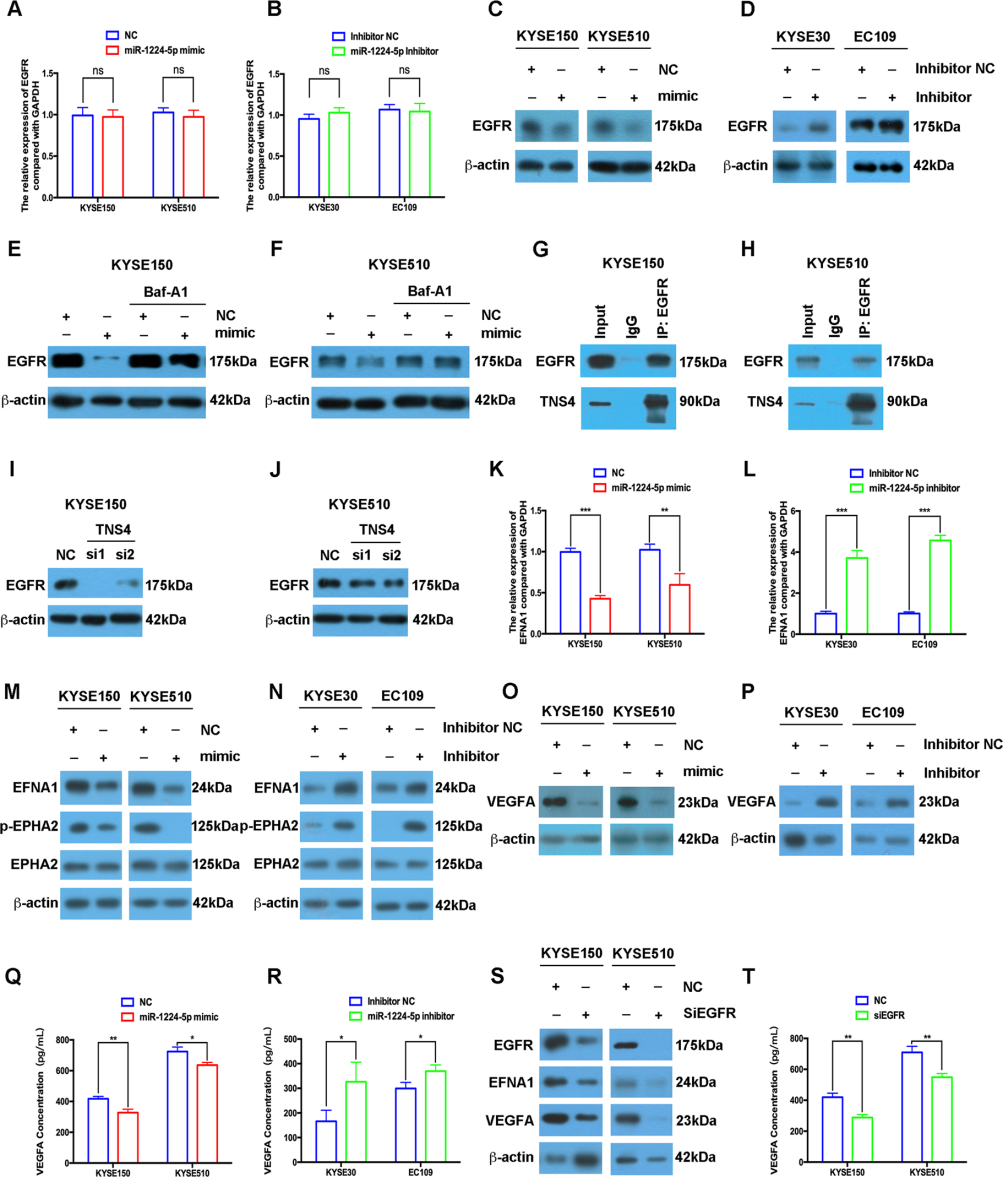
miR-1224-5p regulates the autophagic degradation of EGFR and downstream EFNA1/EPHA2 and VEGFA signalling pathways. **a**–**d** qRT-PCR and western blotting assays were used to examine the mRNA and protein levels of EGFR in ESCC cells transfected with miR-1224-5p mimic or inhibitor. **e**, **f** Western blotting assays were performed to measure EGFR expression in miR-1224-5p mimic-transfected and BafA1 (100 nM)-treated KYSE150 and KYSE510 cells. **g**, **h** A co-IP assay was used to examine the interaction between TNS4 and EGFR in KYSE150 and KYSE510 cells. **i**, **j** Western blotting was used to measure the protein levels of EGFR in TNS4-silenced KYSE150 and KYSE510 cells. **k**–**r** In miR-1224-5p-overexpressing or miR-1224-5p-silenced ESCC cells, qRT-PCR, western blotting and ELISA assays were used to examine the mRNA level of EFNA1 and the protein levels of EFNA1, p-EPHA2, EPHA2, VEGFA and VEGFA in the ESCC cell supernatant. **s**, **t** Western blotting and ELISA assays were performed to measure the protein levels of EGFR, EFNA1, VEGFA and VEGFA in the ESCC cell supernatant after EGFR knockdown. Data are represented as the mean ± SD, and experiments were performed in triplicate; * *p* < 0.05, ** *p* < 0.01, *** *p* < 0.001, ns not significant.

A previous study showed that TNS4 upregulated EGFR protein levels by inhibiting the ubiquitination–proteasome pathway^14^. Therefore, we evaluated whether the miR-1224-5p/TNS4 axis could regulate EGFR in ESCC. Overexpression or silencing of miR-1224-5p had no effects on EGFR mRNA levels in ESCC cells (Fig. 4a, b). Gain of miR-1224-5p decreased EGFR protein levels in KYSE150 and KYSE510 cells, and in contrast, loss of miR-1224-5p increased EGFR expression in KYSE30 and EC109 cells (Fig. 4c, d). Importantly, bafilomycin A1 (BafA1, an inhibitor of autophagic flux) significantly restored the EGFR protein level in miR-1224-5p mimic-transfected KYSE150 and KYSE510 cells compared to control cells (Fig. 4e, f). We further found that TNS4 interacted with EGFR, and knockdown of TNS4 reduced EGFR protein expression (Fig. 4g–j).

EFNA1 and its receptor EPHA2 were upregulated and linked to shorter survival in patients, and activation of the EFNA1/EPHA2 signalling pathway regulated the proliferation and metastasis of oesophageal cancer cells^15–17^. We found that overexpression of miR-1224-5p decreased the mRNA and protein expression of EFNA1 in KYSE150 and KYSE510 cells, and in contrast, miR-1224-5p inhibitors enhanced the expression levels of EFNA1 in KYSE30 and EC109 cells (Fig. 4k–n). Meanwhile, the miR-1224-5p mimic inhibited the phosphorylation of EPHA2, and knockdown of miR-1224-5p had the opposite effects (Fig. 4m, n). Western blotting and ELISA assays further showed that the miR-1224-5p mimic downregulated VEGFA protein levels and decreased VEGFA levels in ESCC cell supernatants, and silencing miR-1224-5p had opposite effects (Fig. 4o–r). We further found that silencing EGFR in KYSE150 and KYSE510 cells significantly decreased the expression of EFNA1 and VEGFA, and reduced VEGFA levels in the supernatant (Fig. 4s, t, Supplementary Fig. 4).

### Knockdown of TNS4 attenuates the oncogenic effects of miR-1224-5p loss in ESCC

**Fig. 5:**
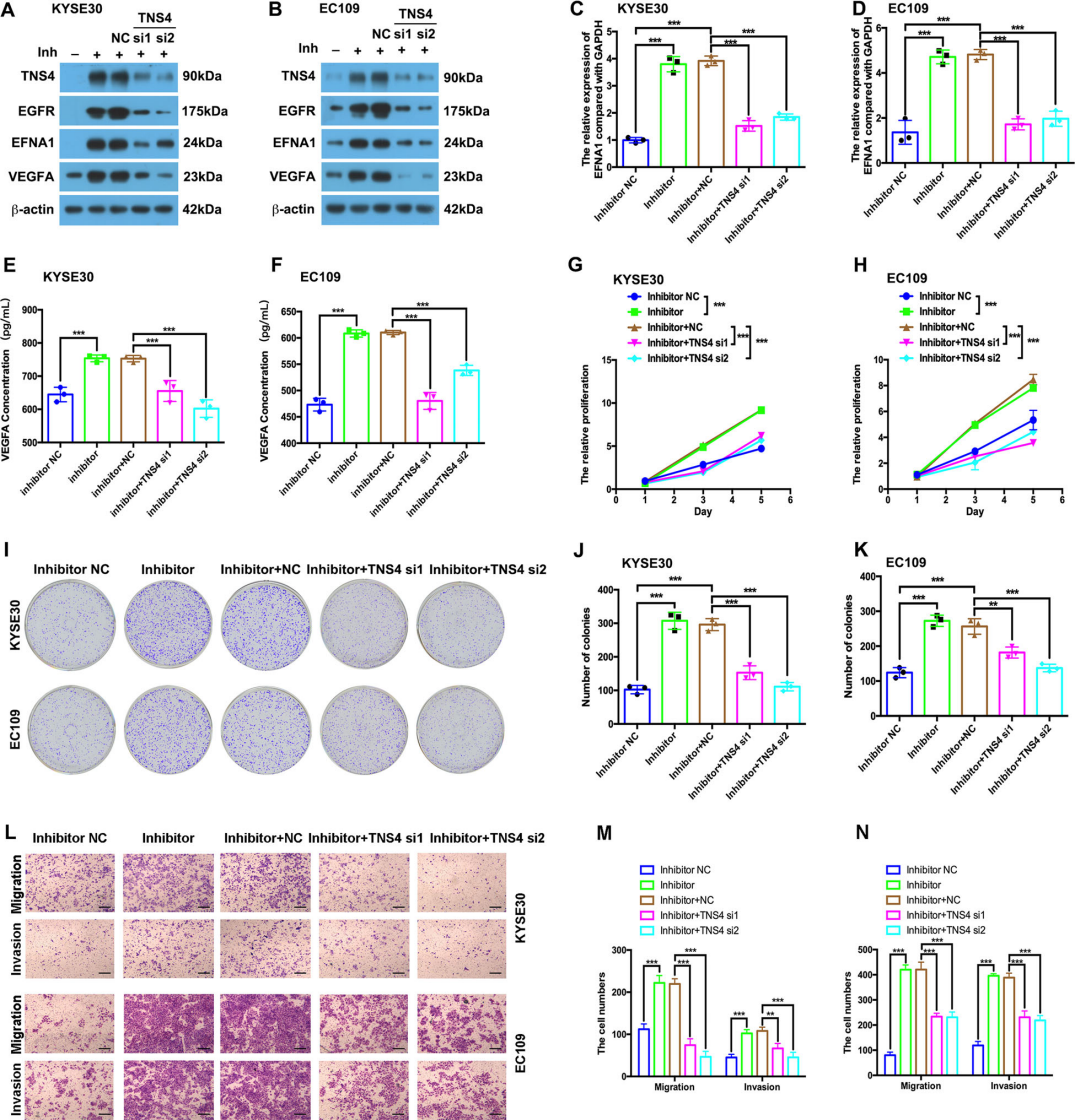
Knockdown of TNS4 rescues the oncogenic roles of miR-1224-5p loss in ESCC. **a**–**f** In KYSE30 and EC109 cells co-transfected with miR-1224-5p inhibitor and TNS4 siRNAs, western blotting, qRT-PCR and ELISA assays were used to analyse the protein levels of TNS4, EGFR, EFNA1 and VEGFA, and the mRNA levels of EFNA1 and VEGFA in the ESCC cell supernatant. **g**–**k** CCK-8 and colony formation assays were used to analyse the proliferation ability of co-transfected cells. **l**–**n** Transwell assays were performed to examine the migration and invasion abilities of co-transfected cells. Scale bar, 200 μm. Data are represented as the mean ± SD, and experiments were performed in triplicate; ** *p* < 0.01; *** *p* < 0.001.

To verify whether knockdown of TNS4 could reverse the effect of miR-1224-5p loss, we co-transfected KYSE30 and EC109 cells with miR-1224-5p inhibitor and TNS4 siRNA. TNS4 expression was decreased after transfection with TNS4 siRNA compared with the negative control in miR-1224-5p inhibitor-transfected cells (Fig. 5a, b). Silencing TNS4 inhibited miR-1224-5p loss-induced upregulation of EGFR, EFNA1 and VEGFA at the protein level (Fig. 5a, b). Knockdown of TNS4 attenuated the miR-1224-5p silencing-induced EFNA1 upregulation at the mRNA level and VEGFA levels in the supernatant (Fig. 5c–f). Silencing TNS4 inhibited miR-1224-5p loss-induced proliferation, colony formation, migration and invasion in both KYSE30 and EC109 cells (Fig. 5g–n).

### Overexpression of miR-1224-5p inhibits tumour growth in vivo

**Fig. 6:**
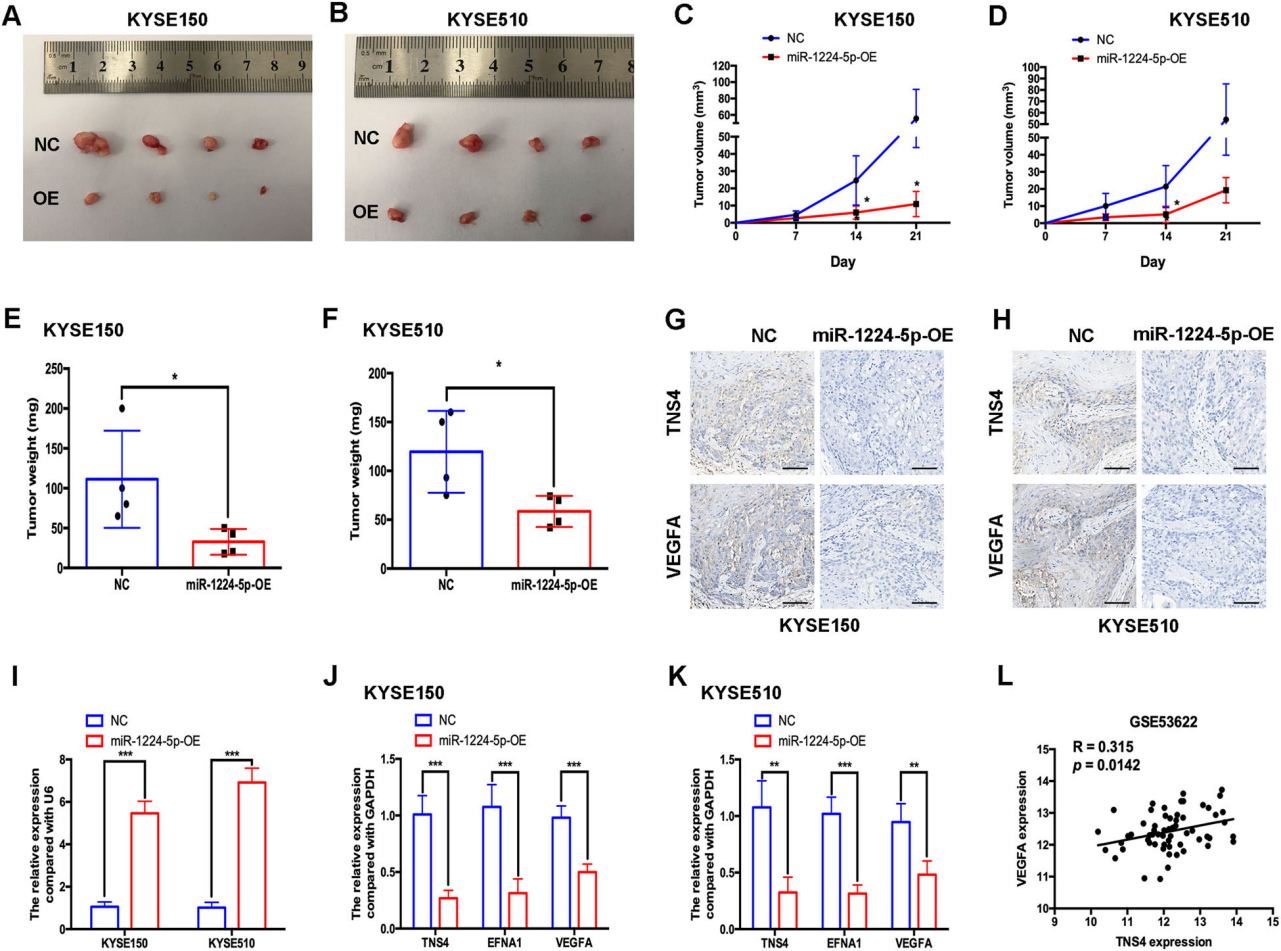
Overexpression of miR-1224-5p inhibits tumour growth *in vivo*. **a**–**f** KYSE150 and KYSE510 cells transfected with negative control vector or miR-1224-5p-OE vector were inoculated into mice subcutaneously. Then, tumour growth, tumour volume and tumour weight were examined in different groups. **g**, **h** Immunohistochemical staining of TNS4 and VEGFA in subcutaneous tumour tissues. Scale bar = 50 μm. **i**–**k** qRT-PCR was used to evaluate the expression of miR-1224-5p, TNS4, EFNA1 and VEGFA in subcutaneous tumour tissues of different groups. **l** The ESCC dataset from GSE53622 was used to analyse the correlation in mRNA level between TNS4 and VEGFA. Data are represented as the mean ± SD; * *p* < 0.05; ** *p* < 0.01; *** *p* < 0.001.

We conducted in vivo experiments to investigate the effect of miR-1224-5p on ESCC tumour growth. We generated KYSE150 and KYSE510 cells stably overexpressing miR-1224-5p by transducing the miR-1224-5p-OE vector. The tumour volume and weight were evaluated every week, and at the end of the experiment, respectively. miR-1224-5p overexpression significantly suppressed the tumour growth of KYSE150 and KYSE510 cells (Fig. 6a–f). IHC analysis confirmed the decrease in TNS4 and VEGFA in the miR-1224-5p-OE group compared with the negative control group (Fig. 6g, h). Using qRT-PCR, we confirmed the overexpression of miR-1224-5p in miR-1224-5p-OE KYSE150 and KYSE510 cells (Fig. 6i). The expression levels of TNS4, EFNA1 and VEGFA were all lower in the miR-1224-5p-OE group than in the negative control group (Fig. 6j, k). The ESCC dataset (GSE53622) further indicated that TNS4 was positively correlated with VEGFA at the mRNA level (Fig. 6l).

### Prognostic significance of TNS4 and VEGFA in ESCC

**Fig. 7:**
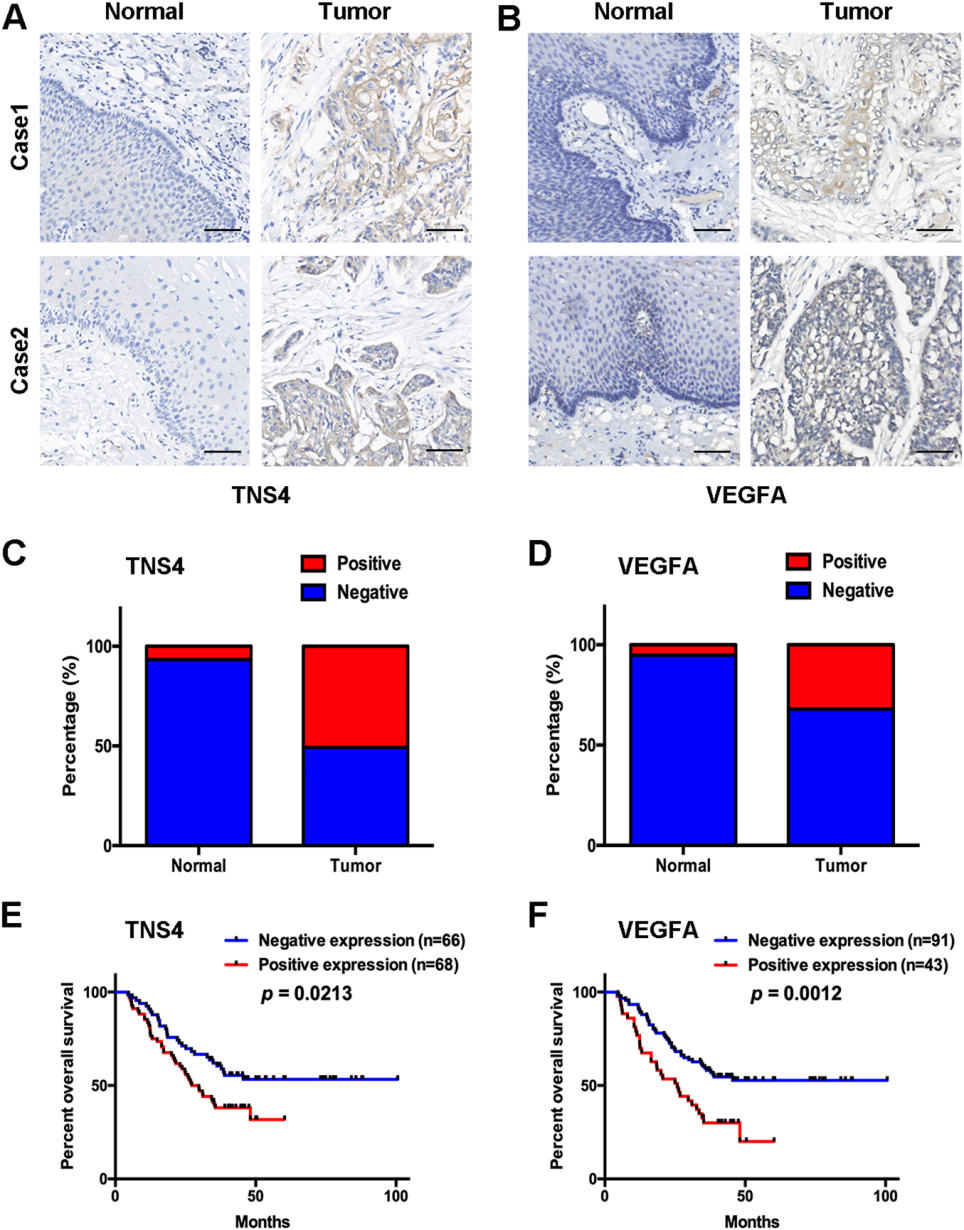
Prognostic significance of TNS4 and VEGFA in ESCC. **a**, **b** Representative immunohistochemical images of TNS4 and VEGFA in tumour samples, and the corresponding normal epithelium. Scale bar = 50 μm. **c**, **d** The positive rates of TNS4 and VEGFA in tumour samples and the corresponding normal epithelium. **e**, **f** Kaplan–Meier curves showing the correlations between TNS4-negative or VEGFA-negative expression in tumours and higher survival rates in patients.

IHC was applied to evaluate the expression and prognostic value of TNS4 and VEGFA in ESCC samples. Positive expression of TNS4 and VEGFA was detected in 6.7% and 5.2% (*n* = 134) of adjacent non-malignant tissues, respectively, and the positive rates of TNS4 and VEGFA in tumour tissues were 50.7% and 32.1% (*n* = 134), respectively (Fig. 7a–d). Spearman’s correlation analysis showed that TNS4-positive expression was significantly associated with the positive expression of VEGFA in ESCC samples (*R* = 0.453, *p* < 0.001, Supplementary Table 6). Importantly, positive expression of TNS4 and VEGFA was significantly associated with lymph node metastasis, and short survival time of ESCC patients, respectively (Fig. 7e, f, Supplementary Table 7).

## Discussion

miRNAs play important roles in oesophageal cancer progression, including tumour growth, metastasis, radiosensitivity and chemoresistance^6,7,18,19^. In addition, miRNAs have shown diagnostic and prognostic value in ESCC. Low miR-1 and high miR-10b-3p expression levels are associated with lymph node metastasis and shorter survival time in patients^20^, and miR-451a, miR-16-5p and miR-574-5p have been identified as diagnostic biomarkers in ESCC^21^. However, the functional roles of many other miRNAs are still unknown in ESCC.

We identified six miRNAs, including let-7c, miR-1224-5p, miR-1226*, miR-320c, miR-630 and miR-939, that are differentially expressed in ESCC samples compared to normal controls. We specifically focused on miR-1224-5p because its roles in cancer, especially squamous cell carcinoma (SCC), are largely unknown. Previous studies reported that miR-1224-5p is positively linked with the overall survival of intestinal-type gastric cancer and glioma patients. Functional studies showed that miR-1224-5p suppresses tumour metastasis by targeting FAK in intestinal-type gastric cancer, and inhibits the proliferation and invasion of glioma cells by targeting CREB1 (refs. ^9,22^). miR-1224-5p, which is sponged by lncRNA ZEB1-AS1, functions as a tumour suppressor in melanoma^23^. However, in SCC, especially ESCC, the function, clinicopathologic correlation and prognostic value of miR-1224-5p are still unknown.

Both our study and a previous study found that miR-1224-5p was downregulated in ESCC^24^. Our present data indicated that its low expression was significantly associated with shorter survival time of ESCC patients.

Our results revealed that miR-1224-5p inhibited the proliferation, colony formation, migration and invasion of ESCC cells by directly targeting TNS4. TNS4 belongs to the tensin protein family and participates in many biological processes, including cell proliferation, migration and adhesion^25^. TNS4 is overexpressed in many types of cancer, such as colorectal cancer, gastric cancer and hepatocellular cancer, and plays an oncogenic role in carcinogenesis^25–27^. To date, the role of TNS4 in ESCC is still unknown. We first reported that TNS4 was upregulated in ESCC at both the mRNA and protein levels, and that its positive expression was significantly associated with a shorter overall survival time in ESCC patients. Silencing TNS4, similar to miR-1224-5p overexpression, significantly inhibited the proliferation, colony formation, migration and invasion of ESCC cells. Previous studies indicated that TNS4 is transcriptionally regulated by the ΔNp63α and MAP kinase signalling pathways, and posttranscriptionally mediated by miR-150-3p (refs. ^25,28,29^). Our findings further provide a new mechanism by that TNS4 is posttranscriptionally regulated by miR-1224-5p in cancer cells.

Our study revealed that miR-1224-5p reduced the protein expression of EGFR, and blocking the autophagic pathway using BafA1 significantly restored the EGFR protein level, which was decreased by the miR-1224-5p mimic. We also found that EGFR interacted with and was regulated by TNS4 in ESCC cells. The autophagic degradation of EGFR is often suppressed in cancer, and activation of this process indicates antitumour activity. Arsenic trioxide circumvents gefitinib resistance by enhancing autophagic degradation of EGFR by directly binding to p62, which interacts with EGFR in NSCLC^30^. Our findings suggest that both gain of miR-1224-5p and loss of TNS4 could downregulate EGFR; however, how the interaction between TNS4 and EGFR participates in regulating the stability of EGFR needs to be explored.

EPHA2 is a member of the ephrin receptor subfamily of protein tyrosine kinases, and EFNA1 (ephrin A1) is its ligand. Recent studies considered the EPHA2 signalling pathway as a target of cancer therapy, and revealed that blocking EPHA2 reversed the acquired resistance of gastric cancer cells to afatinib and inhibit the proliferation of small cell lung cancer cells^31,32^. In ESCC, EFNA1 and EPHA2 were upregulated, and their overexpression was correlated with short overall survival in patients^17,33,34^. EFNA1 conferred resistance to photofrin-mediated photodynamic therapy resistance in ESCC cells, and EPHA2, which was hyperphosphorylated in ESCC, promoted tumour cell proliferation, migration, invasion and epithelial–mesenchymal transition^15,16,35^. VEGFA belongs to the VEGF family and is an important cytokine for angiogenesis^36^. Our results indicated that miR-1224-5p inhibited the EFNA1/EPHA2 and VEGFA signalling pathways. However, the mechanism by that the miR-1224-5p/TNS4/EGFR axis regulates the EFNA1/EPHA2 pathway, and VEGFA needs to be explored in the future.

In conclusion, miR-1224-5p expression is decreased in ESCC compared to normal controls, and low expression of miR-1224-5p is a prognostic marker in ESCC. The findings also indicate that miR-1224-5p inhibits cell proliferation, colony formation, migration and invasion in vitro and tumour growth in vivo by targeting TNS4, and subsequently promoting the autophagic degradation of EGFR, and suppressing downstream EFNA1/EPHA2 and VEGFA signalling pathways. Our study suggests that the miR-1224-5p/TNS4/EGFR axis might be a potential target in ESCC.

## Supplementary information

Table S1

Table S2

Table S3

Table S4

Table S5

Table S6

Table S7

Table S8

Figure S1

Figure S2

Figure S3

Figure S4
