# Supplementary material for: The miR-1224-5p/TNS4/EGFR axis inhibits tumour progression in oesophageal squamous cell carcinoma
Source: Cell Death Dis. 2020 Jul 30;11(7):597. doi: 10.1038/s41419-020-02801-6 (PMC7393493; doi:10.1038/s41419-020-02801-6)
Supplement: Supplementary file 1 — Table S1 [file 41419_2020_2801_MOESM1_ESM.docx]

**Table S1. The information of primers**

| Gene | Forward primer | Reverse primer | Product size |
| --- | --- | --- | --- |
| TNS4 | GCCACACCCTGTACCTGAG | TTCTGCCACTTCCGTTGCTC | 244 |
| EGFR | AGGCACGAGTAACAAGCTCAC | ATGAGGACATAACCAGCCACC | 177 |
| EFNA1 | TCAGGCCCATGACAATCCAC | GTGACCGATGCTATGTAGAACC | 79 |
| GAPDH | AAATCCCATCACCATCTTCCAG | GAGTCCTTCCACGATACCAAAGTTG | 310 |
