# Supplementary material for: The miR-1224-5p/TNS4/EGFR axis inhibits tumour progression in oesophageal squamous cell carcinoma
Source: Cell Death Dis. 2020 Jul 30;11(7):597. doi: 10.1038/s41419-020-02801-6 (PMC7393493; doi:10.1038/s41419-020-02801-6)
Supplement: Supplementary file 2 — Table S2 [file 41419_2020_2801_MOESM2_ESM.docx]

**Table S2. Predicted targets of miR-1224-5p by miRDB**

| NO. | Target rank | Target score | miRNA name | Gene symbol | Gene description |
| --- | --- | --- | --- | --- | --- |
| 1 | 1 | 99 | hsa-miR-1224-5p | ZNF99 | zinc finger protein 99 |
| 2 | 2 | 96 | hsa-miR-1224-5p | MS4A6E | membrane spanning 4-domains A6E |
| 3 | 3 | 95 | hsa-miR-1224-5p | FAM160B1 | family with sequence similarity 160 member B1 |
| 4 | 4 | 95 | hsa-miR-1224-5p | TMEM221 | transmembrane protein 221 |
| 5 | 5 | 95 | hsa-miR-1224-5p | ARHGEF19 | Rho guanine nucleotide exchange factor 19 |
| 6 | 6 | 94 | hsa-miR-1224-5p | GTDC1 | glycosyltransferase like domain containing 1 |
| 7 | 7 | 93 | hsa-miR-1224-5p | KITLG | KIT ligand |
| 8 | 8 | 93 | hsa-miR-1224-5p | TROAP | trophinin associated protein |
| 9 | 9 | 93 | hsa-miR-1224-5p | NEBL | nebulette |
| 10 | 10 | 92 | hsa-miR-1224-5p | CPA6 | carboxypeptidase A6 |
| 11 | 11 | 91 | hsa-miR-1224-5p | FNDC3B | fibronectin type III domain containing 3B |
| 12 | 12 | 91 | hsa-miR-1224-5p | HNRNPU | heterogeneous nuclear ribonucleoprotein U |
| 13 | 13 | 91 | hsa-miR-1224-5p | ZNF257 | zinc finger protein 257 |
| 14 | 14 | 90 | hsa-miR-1224-5p | HBP1 | HMG-box transcription factor 1 |
| 15 | 15 | 90 | hsa-miR-1224-5p | CEP120 | centrosomal protein 120 |
| 16 | 16 | 90 | hsa-miR-1224-5p | BCAT1 | branched chain amino acid transaminase 1 |
| 17 | 17 | 89 | hsa-miR-1224-5p | TNS1 | tensin 1 |
| 18 | 18 | 88 | hsa-miR-1224-5p | PPP1R9B | protein phosphatase 1 regulatory subunit 9B |
| 19 | 19 | 88 | hsa-miR-1224-5p | COQ4 | coenzyme Q4 |
| 20 | 20 | 88 | hsa-miR-1224-5p | TMEM175 | transmembrane protein 175 |
| 21 | 21 | 87 | hsa-miR-1224-5p | SV2A | synaptic vesicle glycoprotein 2A |
| 22 | 22 | 87 | hsa-miR-1224-5p | ZNF208 | zinc finger protein 208 |
| 23 | 23 | 87 | hsa-miR-1224-5p | ZNF664 | zinc finger protein 664 |
| 24 | 24 | 86 | hsa-miR-1224-5p | C12orf40 | chromosome 12 open reading frame 40 |
| 25 | 25 | 86 | hsa-miR-1224-5p | FMNL3 | formin like 3 |
| 26 | 26 | 86 | hsa-miR-1224-5p | CCDC186 | coiled-coil domain containing 186 |
| 27 | 27 | 86 | hsa-miR-1224-5p | TSPAN5 | tetraspanin 5 |
| 28 | 28 | 86 | hsa-miR-1224-5p | MADD | MAP kinase activating death domain |
| 29 | 29 | 86 | hsa-miR-1224-5p | ZNF384 | zinc finger protein 384 |
| 30 | 30 | 85 | hsa-miR-1224-5p | ZDHHC15 | zinc finger DHHC-type containing 15 |
| 31 | 31 | 85 | hsa-miR-1224-5p | ZNF418 | zinc finger protein 418 |
| 32 | 32 | 85 | hsa-miR-1224-5p | PFKFB2 | 6-phosphofructo-2-kinase/fructose-2,6-biphosphatase 2 |
| 33 | 33 | 85 | hsa-miR-1224-5p | ZNF676 | zinc finger protein 676 |
| 34 | 34 | 85 | hsa-miR-1224-5p | OGFOD1 | 2-oxoglutarate and iron dependent oxygenase domain containing 1 |
| 35 | 35 | 85 | hsa-miR-1224-5p | ANKIB1 | ankyrin repeat and IBR domain containing 1 |
| 36 | 36 | 85 | hsa-miR-1224-5p | PRRX1 | paired related homeobox 1 |
| 37 | 37 | 84 | hsa-miR-1224-5p | S100A12 | S100 calcium binding protein A12 |
| 38 | 38 | 84 | hsa-miR-1224-5p | FAM205C | family with sequence similarity 205 member C |
| 39 | 39 | 84 | hsa-miR-1224-5p | KRIT1 | KRIT1, ankyrin repeat containing |
| 40 | 40 | 84 | hsa-miR-1224-5p | GALNT2 | polypeptide N-acetylgalactosaminyltransferase 2 |
| 41 | 41 | 83 | hsa-miR-1224-5p | PRSS16 | serine protease 16 |
| 42 | 42 | 83 | hsa-miR-1224-5p | MDH1B | malate dehydrogenase 1B |
| 43 | 43 | 83 | hsa-miR-1224-5p | LBP | lipopolysaccharide binding protein |
| 44 | 44 | 82 | hsa-miR-1224-5p | KLHL4 | kelch like family member 4 |
| 45 | 45 | 82 | hsa-miR-1224-5p | GABARAPL2 | GABA type A receptor associated protein like 2 |
| 46 | 46 | 82 | hsa-miR-1224-5p | ZDHHC6 | zinc finger DHHC-type containing 6 |
| 47 | 47 | 82 | hsa-miR-1224-5p | NIPA2 | NIPA magnesium transporter 2 |
| 48 | 48 | 82 | hsa-miR-1224-5p | RPE | ribulose-5-phosphate-3-epimerase |
| 49 | 49 | 82 | hsa-miR-1224-5p | KRT73 | keratin 73 |
| 50 | 50 | 82 | hsa-miR-1224-5p | XRCC5 | X-ray repair cross complementing 5 |
| 51 | 51 | 82 | hsa-miR-1224-5p | FAM78B | family with sequence similarity 78 member B |
| 52 | 52 | 81 | hsa-miR-1224-5p | ANTXR1 | ANTXR cell adhesion molecule 1 |
| 53 | 53 | 81 | hsa-miR-1224-5p | PCMT1 | protein-L-isoaspartate (D-aspartate) O-methyltransferase |
| 54 | 54 | 81 | hsa-miR-1224-5p | KIAA1549L | KIAA1549 like |
| 55 | 55 | 81 | hsa-miR-1224-5p | MSI2 | musashi RNA binding protein 2 |
| 56 | 56 | 81 | hsa-miR-1224-5p | ZNF716 | zinc finger protein 716 |
| 57 | 57 | 81 | hsa-miR-1224-5p | UBAP2L | ubiquitin associated protein 2 like |
| 58 | 58 | 81 | hsa-miR-1224-5p | FCRL5 | Fc receptor like 5 |
| 59 | 59 | 81 | hsa-miR-1224-5p | 11-Sep | septin 11 |
| 60 | 60 | 80 | hsa-miR-1224-5p | TRIM11 | tripartite motif containing 11 |
| 61 | 61 | 80 | hsa-miR-1224-5p | PRKAG3 | protein kinase AMP-activated non-catalytic subunit gamma 3 |
| 62 | 62 | 80 | hsa-miR-1224-5p | ZADH2 | zinc binding alcohol dehydrogenase domain containing 2 |
| 63 | 63 | 80 | hsa-miR-1224-5p | RANBP10 | RAN binding protein 10 |
| 64 | 64 | 80 | hsa-miR-1224-5p | CXCL6 | C-X-C motif chemokine ligand 6 |
| 65 | 65 | 80 | hsa-miR-1224-5p | CNGB3 | cyclic nucleotide gated channel beta 3 |
| 66 | 66 | 80 | hsa-miR-1224-5p | ACTL6B | actin like 6B |
| 67 | 67 | 80 | hsa-miR-1224-5p | NOVA1 | NOVA alternative splicing regulator 1 |
| 68 | 68 | 79 | hsa-miR-1224-5p | SH3PXD2B | SH3 and PX domains 2B |
| 69 | 69 | 79 | hsa-miR-1224-5p | GIT2 | GIT ArfGAP 2 |
| 70 | 70 | 79 | hsa-miR-1224-5p | EPSTI1 | epithelial stromal interaction 1 |
| 71 | 71 | 79 | hsa-miR-1224-5p | SLC1A2 | solute carrier family 1 member 2 |
| 72 | 72 | 79 | hsa-miR-1224-5p | ZNF138 | zinc finger protein 138 |
| 73 | 73 | 79 | hsa-miR-1224-5p | DERL2 | derlin 2 |
| 74 | 74 | 78 | hsa-miR-1224-5p | SYT14 | synaptotagmin 14 |
| 75 | 75 | 78 | hsa-miR-1224-5p | RHOQ | ras homolog family member Q |
| 76 | 76 | 78 | hsa-miR-1224-5p | GLUD1 | glutamate dehydrogenase 1 |
| 77 | 77 | 78 | hsa-miR-1224-5p | ASCC1 | activating signal cointegrator 1 complex subunit 1 |
| 78 | 78 | 78 | hsa-miR-1224-5p | RHOBTB3 | Rho related BTB domain containing 3 |
| 79 | 79 | 78 | hsa-miR-1224-5p | NAA50 | N(alpha)-acetyltransferase 50, NatE catalytic subunit |
| 80 | 80 | 78 | hsa-miR-1224-5p | CREB1 | cAMP responsive element binding protein 1 |
| 81 | 81 | 77 | hsa-miR-1224-5p | LOC101927322 | uncharacterized LOC101927322 |
| 82 | 82 | 77 | hsa-miR-1224-5p | DHRS12 | dehydrogenase/reductase 12 |
| 83 | 83 | 77 | hsa-miR-1224-5p | DRP2 | dystrophin related protein 2 |
| 84 | 84 | 77 | hsa-miR-1224-5p | DMRT2 | doublesex and mab-3 related transcription factor 2 |
| 85 | 85 | 77 | hsa-miR-1224-5p | SHISA7 | shisa family member 7 |
| 86 | 86 | 77 | hsa-miR-1224-5p | HECTD4 | HECT domain E3 ubiquitin protein ligase 4 |
| 87 | 87 | 77 | hsa-miR-1224-5p | KLHL14 | kelch like family member 14 |
| 88 | 88 | 77 | hsa-miR-1224-5p | ZNF430 | zinc finger protein 430 |
| 89 | 89 | 77 | hsa-miR-1224-5p | COL11A2 | collagen type XI alpha 2 chain |
| 90 | 90 | 76 | hsa-miR-1224-5p | CTXN2 | cortexin 2 |
| 91 | 91 | 76 | hsa-miR-1224-5p | NSD2 | nuclear receptor binding SET domain protein 2 |
| 92 | 92 | 76 | hsa-miR-1224-5p | LGSN | lengsin, lens protein with glutamine synthetase domain |
| 93 | 93 | 76 | hsa-miR-1224-5p | SMAD2 | SMAD family member 2 |
| 94 | 94 | 76 | hsa-miR-1224-5p | GCNT4 | glucosaminyl (N-acetyl) transferase 4 |
| 95 | 95 | 76 | hsa-miR-1224-5p | TOP1 | DNA topoisomerase I |
| 96 | 96 | 76 | hsa-miR-1224-5p | COPB1 | coatomer protein complex subunit beta 1 |
| 97 | 97 | 76 | hsa-miR-1224-5p | CDHR1 | cadherin related family member 1 |
| 98 | 98 | 76 | hsa-miR-1224-5p | SEPHS1 | selenophosphate synthetase 1 |
| 99 | 99 | 76 | hsa-miR-1224-5p | DNAJB14 | DnaJ heat shock protein family (Hsp40) member B14 |
| 100 | 100 | 75 | hsa-miR-1224-5p | TECPR2 | tectonin beta-propeller repeat containing 2 |
| 101 | 101 | 75 | hsa-miR-1224-5p | AMOT | angiomotin |
| 102 | 102 | 75 | hsa-miR-1224-5p | TMEM266 | transmembrane protein 266 |
| 103 | 103 | 75 | hsa-miR-1224-5p | GPM6B | glycoprotein M6B |
| 104 | 104 | 74 | hsa-miR-1224-5p | ZNF493 | zinc finger protein 493 |
| 105 | 105 | 74 | hsa-miR-1224-5p | NSF | N-ethylmaleimide sensitive factor, vesicle fusing ATPase |
| 106 | 106 | 74 | hsa-miR-1224-5p | EYA4 | EYA transcriptional coactivator and phosphatase 4 |
| 107 | 107 | 74 | hsa-miR-1224-5p | TAOK1 | TAO kinase 1 |
| 108 | 108 | 74 | hsa-miR-1224-5p | PTK2 | protein tyrosine kinase 2 |
| 109 | 109 | 74 | hsa-miR-1224-5p | EIF4E1B | eukaryotic translation initiation factor 4E family member 1B |
| 110 | 110 | 74 | hsa-miR-1224-5p | IPO5 | importin 5 |
| 111 | 111 | 74 | hsa-miR-1224-5p | DLG5 | discs large MAGUK scaffold protein 5 |
| 112 | 112 | 73 | hsa-miR-1224-5p | ERH | ERH, mRNA splicing and mitosis factor |
| 113 | 113 | 73 | hsa-miR-1224-5p | HOXD13 | homeobox D13 |
| 114 | 114 | 73 | hsa-miR-1224-5p | HECA | hdc homolog, cell cycle regulator |
| 115 | 115 | 73 | hsa-miR-1224-5p | KRT72 | keratin 72 |
| 116 | 116 | 73 | hsa-miR-1224-5p | G6PC3 | glucose-6-phosphatase catalytic subunit 3 |
| 117 | 117 | 73 | hsa-miR-1224-5p | WWTR1 | WW domain containing transcription regulator 1 |
| 118 | 118 | 73 | hsa-miR-1224-5p | SIRT6 | sirtuin 6 |
| 119 | 119 | 73 | hsa-miR-1224-5p | HIP1R | huntingtin interacting protein 1 related |
| 120 | 120 | 73 | hsa-miR-1224-5p | FYCO1 | FYVE and coiled-coil domain containing 1 |
| 121 | 121 | 72 | hsa-miR-1224-5p | FNIP1 | folliculin interacting protein 1 |
| 122 | 122 | 72 | hsa-miR-1224-5p | RTL5 | retrotransposon Gag like 5 |
| 123 | 123 | 72 | hsa-miR-1224-5p | NFATC2 | nuclear factor of activated T cells 2 |
| 124 | 124 | 71 | hsa-miR-1224-5p | IPO11 | importin 11 |
| 125 | 125 | 71 | hsa-miR-1224-5p | ZBTB41 | zinc finger and BTB domain containing 41 |
| 126 | 126 | 71 | hsa-miR-1224-5p | FGF1 | fibroblast growth factor 1 |
| 127 | 127 | 71 | hsa-miR-1224-5p | CELF2 | CUGBP Elav-like family member 2 |
| 128 | 128 | 71 | hsa-miR-1224-5p | FGB | fibrinogen beta chain |
| 129 | 129 | 71 | hsa-miR-1224-5p | SLC9A8 | solute carrier family 9 member A8 |
| 130 | 130 | 71 | hsa-miR-1224-5p | LMLN | leishmanolysin like peptidase |
| 131 | 131 | 71 | hsa-miR-1224-5p | KCNB1 | potassium voltage-gated channel subfamily B member 1 |
| 132 | 132 | 71 | hsa-miR-1224-5p | CAPN5 | calpain 5 |
| 133 | 133 | 71 | hsa-miR-1224-5p | DCTN4 | dynactin subunit 4 |
| 134 | 134 | 71 | hsa-miR-1224-5p | UBE2W | ubiquitin conjugating enzyme E2 W |
| 135 | 135 | 71 | hsa-miR-1224-5p | FAM222A | family with sequence similarity 222 member A |
| 136 | 136 | 70 | hsa-miR-1224-5p | DGKK | diacylglycerol kinase kappa |
| 137 | 137 | 70 | hsa-miR-1224-5p | RBBP7 | RB binding protein 7, chromatin remodeling factor |
| 138 | 138 | 70 | hsa-miR-1224-5p | MS4A6A | membrane spanning 4-domains A6A |
| 139 | 139 | 70 | hsa-miR-1224-5p | FBXL7 | F-box and leucine rich repeat protein 7 |
| 140 | 140 | 70 | hsa-miR-1224-5p | THRB | thyroid hormone receptor beta |
| 141 | 141 | 70 | hsa-miR-1224-5p | TUBB2A | tubulin beta 2A class IIa |
| 142 | 142 | 70 | hsa-miR-1224-5p | SAPCD1 | suppressor APC domain containing 1 |
| 143 | 143 | 69 | hsa-miR-1224-5p | RER1 | retention in endoplasmic reticulum sorting receptor 1 |
| 144 | 144 | 69 | hsa-miR-1224-5p | PLCXD3 | phosphatidylinositol specific phospholipase C X domain containing 3 |
| 145 | 145 | 69 | hsa-miR-1224-5p | FKBP5 | FKBP prolyl isomerase 5 |
| 146 | 146 | 69 | hsa-miR-1224-5p | TRIM68 | tripartite motif containing 68 |
| 147 | 147 | 69 | hsa-miR-1224-5p | DCLK1 | doublecortin like kinase 1 |
| 148 | 148 | 69 | hsa-miR-1224-5p | UCHL1 | ubiquitin C-terminal hydrolase L1 |
| 149 | 149 | 69 | hsa-miR-1224-5p | TTC21B | tetratricopeptide repeat domain 21B |
| 150 | 150 | 69 | hsa-miR-1224-5p | RBM41 | RNA binding motif protein 41 |
| 151 | 151 | 69 | hsa-miR-1224-5p | USP24 | ubiquitin specific peptidase 24 |
| 152 | 152 | 69 | hsa-miR-1224-5p | MANBA | mannosidase beta |
| 153 | 153 | 69 | hsa-miR-1224-5p | DOCK1 | dedicator of cytokinesis 1 |
| 154 | 154 | 69 | hsa-miR-1224-5p | TMPRSS11B | transmembrane serine protease 11B |
| 155 | 155 | 68 | hsa-miR-1224-5p | TLN2 | talin 2 |
| 156 | 156 | 68 | hsa-miR-1224-5p | IGF2R | insulin like growth factor 2 receptor |
| 157 | 157 | 68 | hsa-miR-1224-5p | RASD2 | RASD family member 2 |
| 158 | 158 | 68 | hsa-miR-1224-5p | HGSNAT | heparan-alpha-glucosaminide N-acetyltransferase |
| 159 | 159 | 68 | hsa-miR-1224-5p | H3F3B | H3 histone family member 3B |
| 160 | 160 | 68 | hsa-miR-1224-5p | CHCHD3 | coiled-coil-helix-coiled-coil-helix domain containing 3 |
| 161 | 161 | 68 | hsa-miR-1224-5p | USP54 | ubiquitin specific peptidase 54 |
| 162 | 162 | 68 | hsa-miR-1224-5p | ZNF154 | zinc finger protein 154 |
| 163 | 163 | 68 | hsa-miR-1224-5p | XPNPEP3 | X-prolyl aminopeptidase 3 |
| 164 | 164 | 67 | hsa-miR-1224-5p | LCE3D | late cornified envelope 3D |
| 165 | 165 | 67 | hsa-miR-1224-5p | ZNF107 | zinc finger protein 107 |
| 166 | 166 | 67 | hsa-miR-1224-5p | CRYBG1 | crystallin beta-gamma domain containing 1 |
| 167 | 167 | 67 | hsa-miR-1224-5p | ZNF652 | zinc finger protein 652 |
| 168 | 168 | 67 | hsa-miR-1224-5p | AP4B1 | adaptor related protein complex 4 subunit beta 1 |
| 169 | 169 | 67 | hsa-miR-1224-5p | AQP4 | aquaporin 4 |
| 170 | 170 | 67 | hsa-miR-1224-5p | PRICKLE2 | prickle planar cell polarity protein 2 |
| 171 | 171 | 67 | hsa-miR-1224-5p | KLK7 | kallikrein related peptidase 7 |
| 172 | 172 | 67 | hsa-miR-1224-5p | RAB2A | RAB2A, member RAS oncogene family |
| 173 | 173 | 67 | hsa-miR-1224-5p | PITPNM3 | PITPNM family member 3 |
| 174 | 174 | 67 | hsa-miR-1224-5p | CEACAM19 | carcinoembryonic antigen related cell adhesion molecule 19 |
| 175 | 175 | 67 | hsa-miR-1224-5p | BRD8 | bromodomain containing 8 |
| 176 | 176 | 66 | hsa-miR-1224-5p | EXOC2 | exocyst complex component 2 |
| 177 | 177 | 66 | hsa-miR-1224-5p | NPHS2 | NPHS2, podocin |
| 178 | 178 | 66 | hsa-miR-1224-5p | UBXN4 | UBX domain protein 4 |
| 179 | 179 | 66 | hsa-miR-1224-5p | ZFC3H1 | zinc finger C3H1-type containing |
| 180 | 180 | 66 | hsa-miR-1224-5p | KCNMA1 | potassium calcium-activated channel subfamily M alpha 1 |
| 181 | 181 | 66 | hsa-miR-1224-5p | PMEPA1 | prostate transmembrane protein, androgen induced 1 |
| 182 | 182 | 66 | hsa-miR-1224-5p | PKD1 | polycystin 1, transient receptor potential channel interacting |
| 183 | 183 | 66 | hsa-miR-1224-5p | CNNM3 | cyclin and CBS domain divalent metal cation transport mediator 3 |
| 184 | 184 | 66 | hsa-miR-1224-5p | PAPOLA | poly(A) polymerase alpha |
| 185 | 185 | 66 | hsa-miR-1224-5p | ZNF736 | zinc finger protein 736 |
| 186 | 186 | 65 | hsa-miR-1224-5p | ADA2 | adenosine deaminase 2 |
| 187 | 187 | 65 | hsa-miR-1224-5p | CREB5 | cAMP responsive element binding protein 5 |
| 188 | 188 | 65 | hsa-miR-1224-5p | ZNF589 | zinc finger protein 589 |
| 189 | 189 | 65 | hsa-miR-1224-5p | IL1RAP | interleukin 1 receptor accessory protein |
| 190 | 190 | 65 | hsa-miR-1224-5p | SEMA7A | semaphorin 7A (John Milton Hagen blood group) |
| 191 | 191 | 65 | hsa-miR-1224-5p | KIAA1549 | KIAA1549 |
| 192 | 192 | 65 | hsa-miR-1224-5p | MB21D2 | Mab-21 domain containing 2 |
| 193 | 193 | 65 | hsa-miR-1224-5p | WFIKKN2 | WAP, follistatin/kazal, immunoglobulin, kunitz and netrin domain containing 2 |
| 194 | 194 | 65 | hsa-miR-1224-5p | SNX20 | sorting nexin 20 |
| 195 | 195 | 65 | hsa-miR-1224-5p | CLEC4F | C-type lectin domain family 4 member F |
| 196 | 196 | 64 | hsa-miR-1224-5p | FZD4 | frizzled class receptor 4 |
| 197 | 197 | 64 | hsa-miR-1224-5p | NSD1 | nuclear receptor binding SET domain protein 1 |
| 198 | 198 | 64 | hsa-miR-1224-5p | TTC5 | tetratricopeptide repeat domain 5 |
| 199 | 199 | 64 | hsa-miR-1224-5p | HOXC10 | homeobox C10 |
| 200 | 200 | 64 | hsa-miR-1224-5p | CNDP1 | carnosine dipeptidase 1 |
| 201 | 201 | 64 | hsa-miR-1224-5p | UTP25 | UTP25, small subunit processor component |
| 202 | 202 | 64 | hsa-miR-1224-5p | NME9 | NME/NM23 family member 9 |
| 203 | 203 | 64 | hsa-miR-1224-5p | THSD7A | thrombospondin type 1 domain containing 7A |
| 204 | 204 | 63 | hsa-miR-1224-5p | UCK1 | uridine-cytidine kinase 1 |
| 205 | 205 | 63 | hsa-miR-1224-5p | FIGN | fidgetin, microtubule severing factor |
| 206 | 206 | 63 | hsa-miR-1224-5p | ZNF471 | zinc finger protein 471 |
| 207 | 207 | 63 | hsa-miR-1224-5p | TTC3 | tetratricopeptide repeat domain 3 |
| 208 | 208 | 63 | hsa-miR-1224-5p | TMCO1 | transmembrane and coiled-coil domains 1 |
| 209 | 209 | 63 | hsa-miR-1224-5p | NFIB | nuclear factor I B |
| 210 | 210 | 63 | hsa-miR-1224-5p | PLAT | plasminogen activator, tissue type |
| 211 | 211 | 63 | hsa-miR-1224-5p | UBE2QL1 | ubiquitin conjugating enzyme E2 Q family like 1 |
| 212 | 212 | 63 | hsa-miR-1224-5p | SCP2 | sterol carrier protein 2 |
| 213 | 213 | 63 | hsa-miR-1224-5p | LMNB1 | lamin B1 |
| 214 | 214 | 62 | hsa-miR-1224-5p | MSL1 | MSL complex subunit 1 |
| 215 | 215 | 62 | hsa-miR-1224-5p | RNASE3 | ribonuclease A family member 3 |
| 216 | 216 | 62 | hsa-miR-1224-5p | METAP1 | methionyl aminopeptidase 1 |
| 217 | 217 | 62 | hsa-miR-1224-5p | ESYT1 | extended synaptotagmin 1 |
| 218 | 218 | 62 | hsa-miR-1224-5p | ITSN1 | intersectin 1 |
| 219 | 219 | 62 | hsa-miR-1224-5p | SLIT2 | slit guidance ligand 2 |
| 220 | 220 | 62 | hsa-miR-1224-5p | MINDY2 | MINDY lysine 48 deubiquitinase 2 |
| 221 | 221 | 62 | hsa-miR-1224-5p | GJB5 | gap junction protein beta 5 |
| 222 | 222 | 62 | hsa-miR-1224-5p | TRAM2 | translocation associated membrane protein 2 |
| 223 | 223 | 62 | hsa-miR-1224-5p | KLHL7 | kelch like family member 7 |
| 224 | 224 | 62 | hsa-miR-1224-5p | INSR | insulin receptor |
| 225 | 225 | 62 | hsa-miR-1224-5p | RPRD2 | regulation of nuclear pre-mRNA domain containing 2 |
| 226 | 226 | 61 | hsa-miR-1224-5p | CCDC144NL | coiled-coil domain containing 144 family, N-terminal like |
| 227 | 227 | 61 | hsa-miR-1224-5p | TMEM30B | transmembrane protein 30B |
| 228 | 228 | 61 | hsa-miR-1224-5p | ZNF117 | zinc finger protein 117 |
| 229 | 229 | 61 | hsa-miR-1224-5p | UPRT | uracil phosphoribosyltransferase homolog |
| 230 | 230 | 61 | hsa-miR-1224-5p | ZNF343 | zinc finger protein 343 |
| 231 | 231 | 61 | hsa-miR-1224-5p | SND1 | staphylococcal nuclease and tudor domain containing 1 |
| 232 | 232 | 61 | hsa-miR-1224-5p | E2F8 | E2F transcription factor 8 |
| 233 | 233 | 61 | hsa-miR-1224-5p | CD300E | CD300e molecule |
| 234 | 234 | 61 | hsa-miR-1224-5p | PML | promyelocytic leukemia |
| 235 | 235 | 61 | hsa-miR-1224-5p | NUFIP2 | nuclear FMR1 interacting protein 2 |
| 236 | 236 | 61 | hsa-miR-1224-5p | SLC17A6 | solute carrier family 17 member 6 |
| 237 | 237 | 61 | hsa-miR-1224-5p | LILRA1 | leukocyte immunoglobulin like receptor A1 |
| 238 | 238 | 61 | hsa-miR-1224-5p | ZNF449 | zinc finger protein 449 |
| 239 | 239 | 61 | hsa-miR-1224-5p | CDKL5 | cyclin dependent kinase like 5 |
| 240 | 240 | 60 | hsa-miR-1224-5p | SH3RF1 | SH3 domain containing ring finger 1 |
| 241 | 241 | 60 | hsa-miR-1224-5p | SPATA13 | spermatogenesis associated 13 |
| 242 | 242 | 60 | hsa-miR-1224-5p | CD200R1 | CD200 receptor 1 |
| 243 | 243 | 60 | hsa-miR-1224-5p | B3GLCT | beta 3-glucosyltransferase |
| 244 | 244 | 60 | hsa-miR-1224-5p | ZMAT4 | zinc finger matrin-type 4 |
| 245 | 245 | 60 | hsa-miR-1224-5p | TNNC1 | troponin C1, slow skeletal and cardiac type |
| 246 | 246 | 59 | hsa-miR-1224-5p | TLDC2 | TBC/LysM-associated domain containing 2 |
| 247 | 247 | 59 | hsa-miR-1224-5p | RUNX1T1 | RUNX1 translocation partner 1 |
| 248 | 248 | 59 | hsa-miR-1224-5p | DES | desmin |
| 249 | 249 | 59 | hsa-miR-1224-5p | SLA2 | Src like adaptor 2 |
| 250 | 250 | 59 | hsa-miR-1224-5p | PHF6 | PHD finger protein 6 |
| 251 | 251 | 59 | hsa-miR-1224-5p | IGFALS | insulin like growth factor binding protein acid labile subunit |
| 252 | 252 | 59 | hsa-miR-1224-5p | HPSE | heparanase |
| 253 | 253 | 59 | hsa-miR-1224-5p | PDK2 | pyruvate dehydrogenase kinase 2 |
| 254 | 254 | 58 | hsa-miR-1224-5p | LOC100132813 | uncharacterized LOC100132813 |
| 255 | 255 | 58 | hsa-miR-1224-5p | HVCN1 | hydrogen voltage gated channel 1 |
| 256 | 256 | 58 | hsa-miR-1224-5p | ERC2 | ELKS/RAB6-interacting/CAST family member 2 |
| 257 | 257 | 58 | hsa-miR-1224-5p | EPM2A | EPM2A, laforin glucan phosphatase |
| 258 | 258 | 58 | hsa-miR-1224-5p | ACVR2B | activin A receptor type 2B |
| 259 | 259 | 58 | hsa-miR-1224-5p | SKP1 | S-phase kinase associated protein 1 |
| 260 | 260 | 58 | hsa-miR-1224-5p | UBE2V1 | ubiquitin conjugating enzyme E2 V1 |
| 261 | 261 | 58 | hsa-miR-1224-5p | FADS1 | fatty acid desaturase 1 |
| 262 | 262 | 58 | hsa-miR-1224-5p | CENPO | centromere protein O |
| 263 | 263 | 58 | hsa-miR-1224-5p | DYRK1A | dual specificity tyrosine phosphorylation regulated kinase 1A |
| 264 | 264 | 58 | hsa-miR-1224-5p | INAVA | innate immunity activator |
| 265 | 265 | 58 | hsa-miR-1224-5p | LUZP1 | leucine zipper protein 1 |
| 266 | 266 | 58 | hsa-miR-1224-5p | MAP7D3 | MAP7 domain containing 3 |
| 267 | 267 | 58 | hsa-miR-1224-5p | SUN1 | Sad1 and UNC84 domain containing 1 |
| 268 | 268 | 58 | hsa-miR-1224-5p | MED20 | mediator complex subunit 20 |
| 269 | 269 | 58 | hsa-miR-1224-5p | FUNDC2 | FUN14 domain containing 2 |
| 270 | 270 | 58 | hsa-miR-1224-5p | APOLD1 | apolipoprotein L domain containing 1 |
| 271 | 271 | 58 | hsa-miR-1224-5p | KMT5B | lysine methyltransferase 5B |
| 272 | 272 | 57 | hsa-miR-1224-5p | ARHGAP29 | Rho GTPase activating protein 29 |
| 273 | 273 | 57 | hsa-miR-1224-5p | ITGAV | integrin subunit alpha V |
| 274 | 274 | 57 | hsa-miR-1224-5p | ENSA | endosulfine alpha |
| 275 | 275 | 57 | hsa-miR-1224-5p | CUL3 | cullin 3 |
| 276 | 276 | 57 | hsa-miR-1224-5p | C10orf67 | chromosome 10 open reading frame 67 |
| 277 | 277 | 57 | hsa-miR-1224-5p | KLF3 | Kruppel like factor 3 |
| 278 | 278 | 57 | hsa-miR-1224-5p | KPNA1 | karyopherin subunit alpha 1 |
| 279 | 279 | 57 | hsa-miR-1224-5p | ATOH7 | atonal bHLH transcription factor 7 |
| 280 | 280 | 57 | hsa-miR-1224-5p | ZNF629 | zinc finger protein 629 |
| 281 | 281 | 57 | hsa-miR-1224-5p | GXYLT1 | glucoside xylosyltransferase 1 |
| 282 | 282 | 57 | hsa-miR-1224-5p | PSEN1 | presenilin 1 |
| 283 | 283 | 57 | hsa-miR-1224-5p | RNASE2 | ribonuclease A family member 2 |
| 284 | 284 | 56 | hsa-miR-1224-5p | RNF215 | ring finger protein 215 |
| 285 | 285 | 56 | hsa-miR-1224-5p | FUT9 | fucosyltransferase 9 |
| 286 | 286 | 56 | hsa-miR-1224-5p | FAM217B | family with sequence similarity 217 member B |
| 287 | 287 | 56 | hsa-miR-1224-5p | DNAAF3 | dynein axonemal assembly factor 3 |
| 288 | 288 | 56 | hsa-miR-1224-5p | GMFG | glia maturation factor gamma |
| 289 | 289 | 56 | hsa-miR-1224-5p | ADGRL2 | adhesion G protein-coupled receptor L2 |
| 290 | 290 | 56 | hsa-miR-1224-5p | MED11 | mediator complex subunit 11 |
| 291 | 291 | 56 | hsa-miR-1224-5p | DVL3 | dishevelled segment polarity protein 3 |
| 292 | 292 | 56 | hsa-miR-1224-5p | HGS | hepatocyte growth factor-regulated tyrosine kinase substrate |
| 293 | 293 | 56 | hsa-miR-1224-5p | ZNF75A | zinc finger protein 75a |
| 294 | 294 | 56 | hsa-miR-1224-5p | KMT2A | lysine methyltransferase 2A |
| 295 | 295 | 56 | hsa-miR-1224-5p | ARHGEF9 | Cdc42 guanine nucleotide exchange factor 9 |
| 296 | 296 | 56 | hsa-miR-1224-5p | CDIP1 | cell death inducing p53 target 1 |
| 297 | 297 | 56 | hsa-miR-1224-5p | KRTAP8-1 | keratin associated protein 8-1 |
| 298 | 298 | 56 | hsa-miR-1224-5p | HTRA3 | HtrA serine peptidase 3 |
| 299 | 299 | 56 | hsa-miR-1224-5p | MAP3K13 | mitogen-activated protein kinase kinase kinase 13 |
| 300 | 300 | 56 | hsa-miR-1224-5p | TEAD3 | TEA domain transcription factor 3 |
| 301 | 301 | 55 | hsa-miR-1224-5p | MNT | MAX network transcriptional repressor |
| 302 | 302 | 55 | hsa-miR-1224-5p | ZNF730 | zinc finger protein 730 |
| 303 | 303 | 55 | hsa-miR-1224-5p | STK16 | serine/threonine kinase 16 |
| 304 | 304 | 55 | hsa-miR-1224-5p | CDK12 | cyclin dependent kinase 12 |
| 305 | 305 | 55 | hsa-miR-1224-5p | LMO7DN | LMO7 downstream neighbor |
| 306 | 306 | 55 | hsa-miR-1224-5p | SSH2 | slingshot protein phosphatase 2 |
| 307 | 307 | 55 | hsa-miR-1224-5p | CBL | Cbl proto-oncogene |
| 308 | 308 | 55 | hsa-miR-1224-5p | SLC46A1 | solute carrier family 46 member 1 |
| 309 | 309 | 55 | hsa-miR-1224-5p | ZNF230 | zinc finger protein 230 |
| 310 | 310 | 55 | hsa-miR-1224-5p | DLX1 | distal-less homeobox 1 |
| 311 | 311 | 55 | hsa-miR-1224-5p | LSAMP | limbic system associated membrane protein |
| 312 | 312 | 55 | hsa-miR-1224-5p | RIMS4 | regulating synaptic membrane exocytosis 4 |
| 313 | 313 | 55 | hsa-miR-1224-5p | GSTM5 | glutathione S-transferase mu 5 |
| 314 | 314 | 54 | hsa-miR-1224-5p | ST8SIA3 | ST8 alpha-N-acetyl-neuraminide alpha-2,8-sialyltransferase 3 |
| 315 | 315 | 54 | hsa-miR-1224-5p | SYNPO2 | synaptopodin 2 |
| 316 | 316 | 54 | hsa-miR-1224-5p | SORCS2 | sortilin related VPS10 domain containing receptor 2 |
| 317 | 317 | 54 | hsa-miR-1224-5p | BAAT | bile acid-CoA:amino acid N-acyltransferase |
| 318 | 318 | 54 | hsa-miR-1224-5p | ABLIM1 | actin binding LIM protein 1 |
| 319 | 319 | 54 | hsa-miR-1224-5p | LPIN2 | lipin 2 |
| 320 | 320 | 54 | hsa-miR-1224-5p | XPO1 | exportin 1 |
| 321 | 321 | 54 | hsa-miR-1224-5p | KCNE3 | potassium voltage-gated channel subfamily E regulatory subunit 3 |
| 322 | 322 | 54 | hsa-miR-1224-5p | ZHX1 | zinc fingers and homeoboxes 1 |
| 323 | 323 | 54 | hsa-miR-1224-5p | KCMF1 | potassium channel modulatory factor 1 |
| 324 | 324 | 54 | hsa-miR-1224-5p | FMN1 | formin 1 |
| 325 | 325 | 54 | hsa-miR-1224-5p | ITK | IL2 inducible T cell kinase |
| 326 | 326 | 54 | hsa-miR-1224-5p | ACAP3 | ArfGAP with coiled-coil, ankyrin repeat and PH domains 3 |
| 327 | 327 | 54 | hsa-miR-1224-5p | PRC1 | protein regulator of cytokinesis 1 |
| 328 | 328 | 54 | hsa-miR-1224-5p | ANGPT4 | angiopoietin 4 |
| 329 | 329 | 54 | hsa-miR-1224-5p | GRIN2A | glutamate ionotropic receptor NMDA type subunit 2A |
| 330 | 330 | 54 | hsa-miR-1224-5p | MAPK1 | mitogen-activated protein kinase 1 |
| 331 | 331 | 53 | hsa-miR-1224-5p | KLHL15 | kelch like family member 15 |
| 332 | 332 | 53 | hsa-miR-1224-5p | ZNF276 | zinc finger protein 276 |
| 333 | 333 | 53 | hsa-miR-1224-5p | PCDHA9 | protocadherin alpha 9 |
| 334 | 334 | 53 | hsa-miR-1224-5p | CCPG1 | cell cycle progression 1 |
| 335 | 335 | 53 | hsa-miR-1224-5p | TPP1 | tripeptidyl peptidase 1 |
| 336 | 336 | 53 | hsa-miR-1224-5p | MYBPHL | myosin binding protein H like |
| 337 | 337 | 53 | hsa-miR-1224-5p | CRNN | cornulin |
| 338 | 338 | 53 | hsa-miR-1224-5p | SEMA3C | semaphorin 3C |
| 339 | 339 | 53 | hsa-miR-1224-5p | AQP2 | aquaporin 2 |
| 340 | 340 | 53 | hsa-miR-1224-5p | ARL17A | ADP ribosylation factor like GTPase 17A |
| 341 | 341 | 53 | hsa-miR-1224-5p | CHURC1 | churchill domain containing 1 |
| 342 | 342 | 53 | hsa-miR-1224-5p | PSME4 | proteasome activator subunit 4 |
| 343 | 343 | 53 | hsa-miR-1224-5p | CYTH2 | cytohesin 2 |
| 344 | 344 | 53 | hsa-miR-1224-5p | NRP1 | neuropilin 1 |
| 345 | 345 | 53 | hsa-miR-1224-5p | HIST1H2BK | histone cluster 1 H2B family member k |
| 346 | 346 | 53 | hsa-miR-1224-5p | CD160 | CD160 molecule |
| 347 | 347 | 53 | hsa-miR-1224-5p | CERS3 | ceramide synthase 3 |
| 348 | 348 | 53 | hsa-miR-1224-5p | SH3RF2 | SH3 domain containing ring finger 2 |
| 349 | 349 | 53 | hsa-miR-1224-5p | DCUN1D4 | defective in cullin neddylation 1 domain containing 4 |
| 350 | 350 | 53 | hsa-miR-1224-5p | NPL | N-acetylneuraminate pyruvate lyase |
| 351 | 351 | 52 | hsa-miR-1224-5p | INSL3 | insulin like 3 |
| 352 | 352 | 52 | hsa-miR-1224-5p | DPF3 | double PHD fingers 3 |
| 353 | 353 | 52 | hsa-miR-1224-5p | ANK3 | ankyrin 3 |
| 354 | 354 | 52 | hsa-miR-1224-5p | ZNF728 | zinc finger protein 728 |
| 355 | 355 | 52 | hsa-miR-1224-5p | CCDC149 | coiled-coil domain containing 149 |
| 356 | 356 | 52 | hsa-miR-1224-5p | TMEM185B | transmembrane protein 185B |
| 357 | 357 | 52 | hsa-miR-1224-5p | PFKFB1 | 6-phosphofructo-2-kinase/fructose-2,6-biphosphatase 1 |
| 358 | 358 | 52 | hsa-miR-1224-5p | SLC29A4 | solute carrier family 29 member 4 |
| 359 | 359 | 52 | hsa-miR-1224-5p | PANK1 | pantothenate kinase 1 |
| 360 | 360 | 52 | hsa-miR-1224-5p | CPNE1 | copine 1 |
| 361 | 361 | 52 | hsa-miR-1224-5p | PPFIBP1 | PPFIA binding protein 1 |
| 362 | 362 | 52 | hsa-miR-1224-5p | KRT76 | keratin 76 |
| 363 | 363 | 52 | hsa-miR-1224-5p | DLEC1 | DLEC1, cilia and flagella associated protein |
| 364 | 364 | 51 | hsa-miR-1224-5p | TBC1D7 | TBC1 domain family member 7 |
| 365 | 365 | 51 | hsa-miR-1224-5p | SLC25A22 | solute carrier family 25 member 22 |
| 366 | 366 | 51 | hsa-miR-1224-5p | STX6 | syntaxin 6 |
| 367 | 367 | 51 | hsa-miR-1224-5p | SMARCC1 | SWI/SNF related, matrix associated, actin dependent regulator of chromatin subfamily c member 1 |
| 368 | 368 | 51 | hsa-miR-1224-5p | ADORA3 | adenosine A3 receptor |
| 369 | 369 | 51 | hsa-miR-1224-5p | PPP3R2 | protein phosphatase 3 regulatory subunit B, beta |
| 370 | 370 | 51 | hsa-miR-1224-5p | MYO1D | myosin ID |
| 371 | 371 | 51 | hsa-miR-1224-5p | CLDN19 | claudin 19 |
| 372 | 372 | 51 | hsa-miR-1224-5p | LCA5 | LCA5, lebercilin |
| 373 | 373 | 51 | hsa-miR-1224-5p | TRIM41 | tripartite motif containing 41 |
| 374 | 374 | 51 | hsa-miR-1224-5p | C1orf74 | chromosome 1 open reading frame 74 |
| 375 | 375 | 51 | hsa-miR-1224-5p | NEUROD4 | neuronal differentiation 4 |
| 376 | 376 | 51 | hsa-miR-1224-5p | NID1 | nidogen 1 |
| 377 | 377 | 51 | hsa-miR-1224-5p | MGAT4A | alpha-1,3-mannosyl-glycoprotein 4-beta-N-acetylglucosaminyltransferase A |
| 378 | 378 | 51 | hsa-miR-1224-5p | ANKLE1 | ankyrin repeat and LEM domain containing 1 |
| 379 | 379 | 51 | hsa-miR-1224-5p | IYD | iodotyrosine deiodinase |
| 380 | 380 | 51 | hsa-miR-1224-5p | NFYB | nuclear transcription factor Y subunit beta |
| 381 | 381 | 50 | hsa-miR-1224-5p | CNGA3 | cyclic nucleotide gated channel alpha 3 |
| 382 | 382 | 50 | hsa-miR-1224-5p | C16orf89 | chromosome 16 open reading frame 89 |
| 383 | 383 | 50 | hsa-miR-1224-5p | SATB1 | SATB homeobox 1 |
| 384 | 384 | 50 | hsa-miR-1224-5p | MOCS2 | molybdenum cofactor synthesis 2 |
| 385 | 385 | 50 | hsa-miR-1224-5p | STEAP3 | STEAP3 metalloreductase |
| 386 | 386 | 50 | hsa-miR-1224-5p | OGFRL1 | opioid growth factor receptor like 1 |
| 387 | 387 | 50 | hsa-miR-1224-5p | ZCCHC14 | zinc finger CCHC-type containing 14 |
| 388 | 388 | 50 | hsa-miR-1224-5p | DDX3Y | DEAD-box helicase 3 Y-linked |
| 389 | 389 | 50 | hsa-miR-1224-5p | FAM122C | family with sequence similarity 122C |
| 390 | 390 | 50 | hsa-miR-1224-5p | SLC34A1 | solute carrier family 34 member 1 |
| 391 | 391 | 50 | hsa-miR-1224-5p | S1PR1 | sphingosine-1-phosphate receptor 1 |
| 392 | 392 | 50 | hsa-miR-1224-5p | SLCO5A1 | solute carrier organic anion transporter family member 5A1 |
| 393 | 393 | 50 | hsa-miR-1224-5p | TRDN | triadin |
| 394 | 394 | 50 | hsa-miR-1224-5p | CLNS1A | chloride nucleotide-sensitive channel 1A |
| 395 | 395 | 50 | hsa-miR-1224-5p | VPS36 | vacuolar protein sorting 36 homolog |
| 396 | 396 | 50 | hsa-miR-1224-5p | VPS72 | vacuolar protein sorting 72 homolog |
| 397 | 397 | 50 | hsa-miR-1224-5p | TNS4 | tensin 4 |
| 398 | 398 | 50 | hsa-miR-1224-5p | PDK3 | pyruvate dehydrogenase kinase 3 |
| 399 | 399 | 50 | hsa-miR-1224-5p | NOS1 | nitric oxide synthase 1 |
| 400 | 400 | 50 | hsa-miR-1224-5p | PKD2L2 | polycystin 2 like 2, transient receptor potential cation channel |
