# Supplementary material for: The miR-1224-5p/TNS4/EGFR axis inhibits tumour progression in oesophageal squamous cell carcinoma
Source: Cell Death Dis. 2020 Jul 30;11(7):597. doi: 10.1038/s41419-020-02801-6 (PMC7393493; doi:10.1038/s41419-020-02801-6)
Supplement: Supplementary file 3 — Table S3 [file 41419_2020_2801_MOESM3_ESM.docx]

**Table S3. Overexpressed genes in ESCC identified by microarray**

| NO. | Probe name | Fold change  ([T] vs [N]) | Log fold change  ([T] vs [N]) | Absolute fold change  ([T] vs [N]) | Regulation  ([T] vs [N]) | Gene symbol |
| --- | --- | --- | --- | --- | --- | --- |
| 1 | A_23_P37545 | 4.259811 | 2.0907893 | 4.259811 | up | AAGAB |
| 2 | A_23_P5441 | 6.2619433 | 2.6466105 | 6.2619433 | up | ABCB6 |
| 3 | A_23_P258221 | 15.726776 | 3.975151 | 15.726776 | up | ABCC5 |
| 4 | A_23_P315933 | 8.407382 | 3.0716567 | 8.407382 | up | ABHD11 |
| 5 | A_23_P132405 | 7.1408076 | 2.8360872 | 7.1408076 | up | ACAD9 |
| 6 | A_23_P207650 | 5.4300947 | 2.4409773 | 5.4300947 | up | ACADVL |
| 7 | A_33_P3398564 | 10.330714 | 3.368868 | 10.330714 | up | ACAP3 |
| 8 | A_23_P69249 | 21.677343 | 4.438116 | 21.677343 | up | ACTL6A |
| 9 | A_23_P67864 | 11.493751 | 3.5227778 | 11.493751 | up | ADCY3 |
| 10 | A_33_P3384392 | 11.781016 | 3.558392 | 11.781016 | up | ADRBK1 |
| 11 | A_33_P3360540 | 15.70754 | 3.9733853 | 15.70754 | up | AGPAT2 |
| 12 | A_33_P3666346 | 4.954335 | 2.3086915 | 4.954335 | up | AHCTF1 |
| 13 | A_23_P115331 | 2.6603885 | 1.411637 | 2.6603885 | up | AHDC1 |
| 14 | A_33_P3316800 | 8.5093975 | 3.089057 | 8.5093975 | up | AHR |
| 15 | A_24_P179903 | 7.9755106 | 2.9955769 | 7.9755106 | up | AK2 |
| 16 | A_24_P414269 | 24.062357 | 4.588706 | 24.062357 | up | ALG3 |
| 17 | A_33_P3491294 | 4.1133957 | 2.04033 | 4.1133957 | up | AMDHD2 |
| 18 | A_24_P401768 | 2.5842407 | 1.3697405 | 2.5842407 | up | AMOTL1 |
| 19 | A_33_P3211153 | 5.4784465 | 2.4537668 | 5.4784465 | up | ANKS3 |
| 20 | A_33_P3299279 | 2.4782493 | 1.3093213 | 2.4782493 | up | ANXA2R |
| 21 | A_24_P932418 | 4.13265 | 2.0470672 | 4.13265 | up | AP2A2 |
| 22 | A_33_P3215487 | 5.0565214 | 2.3381453 | 5.0565214 | up | AP5Z1 |
| 23 | A_24_P48898 | 15.657236 | 3.9687576 | 15.657236 | up | APOL2 |
| 24 | A_33_P3296479 | 9.125503 | 3.189904 | 9.125503 | up | APP |
| 25 | A_33_P3360097 | 5.297623 | 2.4053452 | 5.297623 | up | APRT |
| 26 | A_33_P3242748 | 8.880237 | 3.150598 | 8.880237 | up | ARFRP1 |
| 27 | A_33_P3339375 | 5.902567 | 2.5613425 | 5.902567 | up | ARHGAP11B |
| 28 | A_33_P3221563 | 11.627392 | 3.5394557 | 11.627392 | up | ARMC5 |
| 29 | A_33_P3228573 | 3.9725552 | 1.9900672 | 3.9725552 | up | ASPSCR1 |
| 30 | A_33_P3331588 | 26.865833 | 4.7477007 | 26.865833 | up | ATAD3B |
| 31 | A_33_P3394040 | 12.033075 | 3.5889335 | 12.033075 | up | ATL2 |
| 32 | A_23_P11353 | 5.4790387 | 2.4539227 | 5.4790387 | up | ATP6AP2 |
| 33 | A_23_P146058 | 4.936451 | 2.3034742 | 4.936451 | up | ATP6V1C1 |
| 34 | A_23_P208389 | 6.379816 | 2.6735148 | 6.379816 | up | AXL |
| 35 | A_33_P3407042 | 4.208846 | 2.0734248 | 4.208846 | up | B3GALT6 |
| 36 | A_33_P3398526 | 14.209135 | 3.8287468 | 14.209135 | up | BCL2L11 |
| 37 | A_33_P3217123 | 5.789864 | 2.5335295 | 5.789864 | up | BCL2L12 |
| 38 | A_23_P118815 | 12.095939 | 3.5964508 | 12.095939 | up | BIRC5 |
| 39 | A_33_P3329597 | 4.050004 | 2.0179234 | 4.050004 | up | BLOC1S3 |
| 40 | A_23_P251893 | 7.8748503 | 2.9772525 | 7.8748503 | up | BRAT1 |
| 41 | A_23_P87560 | 6.7673016 | 2.7585807 | 6.7673016 | up | BTG1 |
| 42 | A_33_P3216237 | 14.174825 | 3.825259 | 14.174825 | up | BZW2 |
| 43 | A_33_P3256858 | 12.9984255 | 3.700265 | 12.9984255 | up | C14orf80 |
| 44 | A_23_P48581 | 2.5733116 | 1.3636261 | 2.5733116 | up | C14orf93 |
| 45 | A_33_P3417195 | 3.9321165 | 1.975306 | 3.9321165 | up | C17orf82 |
| 46 | A_33_P3839760 | 8.540964 | 3.094399 | 8.540964 | up | C19orf24 |
| 47 | A_33_P3288754 | 6.4786363 | 2.6956902 | 6.4786363 | up | C19orf48 |
| 48 | A_23_P56590 | 5.8372498 | 2.5452888 | 5.8372498 | up | C1D |
| 49 | A_33_P3218625 | 5.6913576 | 2.5087729 | 5.6913576 | up | CACFD1 |
| 50 | A_24_P84428 | 9.57501 | 3.259274 | 9.57501 | up | CACYBP |
| 51 | A_23_P31315 | 7.0206227 | 2.811599 | 7.0206227 | up | CBX3 |
| 52 | A_23_P101461 | 9.032124 | 3.1750653 | 9.032124 | up | CCDC130 |
| 53 | A_33_P3359753 | 3.4192235 | 1.7736688 | 3.4192235 | up | CCSAP |
| 54 | A_33_P3229196 | 5.2434993 | 2.3905299 | 5.2434993 | up | CD151 |
| 55 | A_33_P3222917 | 14.786611 | 3.8862195 | 14.786611 | up | CD276 |
| 56 | A_23_P253052 | 6.960049 | 2.7990975 | 6.960049 | up | CD99L2 |
| 57 | A_23_P104651 | 10.539609 | 3.3977494 | 10.539609 | up | CDCA5 |
| 58 | A_23_P138507 | 5.2834516 | 2.4014807 | 5.2834516 | up | CDK1 |
| 59 | A_33_P3230688 | 20.137262 | 4.3317957 | 20.137262 | up | CDK10 |
| 60 | A_33_P3227264 | 5.5026765 | 2.4601336 | 5.5026765 | up | CDK11A |
| 61 | A_33_P3213752 | 5.7352853 | 2.5198653 | 5.7352853 | up | CDK5RAP1 |
| 62 | A_24_P292964 | 2.960256 | 1.565722 | 2.960256 | up | CDK5RAP3 |
| 63 | A_33_P3386262 | 4.50381 | 2.171146 | 4.50381 | up | CDT1 |
| 64 | A_23_P119964 | 4.582943 | 2.1962743 | 4.582943 | up | CEBPZ |
| 65 | A_23_P132378 | 6.0857706 | 2.60544 | 6.0857706 | up | CELSR1 |
| 66 | A_23_P88740 | 4.6192656 | 2.2076635 | 4.6192656 | up | CENPN |
| 67 | A_23_P319270 | 15.97036 | 3.997325 | 15.97036 | up | CEP131 |
| 68 | A_33_P3244863 | 3.092486 | 1.628767 | 3.092486 | up | CEP350 |
| 69 | A_23_P307400 | 10.854069 | 3.440164 | 10.854069 | up | CEP95 |
| 70 | A_24_P53519 | 2.3412545 | 1.2272817 | 2.3412545 | up | CHAF1A |
| 71 | A_33_P3239287 | 4.001283 | 2.0004628 | 4.001283 | up | CHD3 |
| 72 | A_23_P16139 | 3.8743374 | 1.9539496 | 3.8743374 | up | CHERP |
| 73 | A_33_P3316878 | 10.30011 | 3.3645878 | 10.30011 | up | CHPF |
| 74 | A_33_P3412149 | 2.0258763 | 1.0185461 | 2.0258763 | up | CLCC1 |
| 75 | A_33_P3287685 | 3.926502 | 1.9732447 | 3.926502 | up | CNOT3 |
| 76 | A_24_P331904 | 8.265292 | 3.0470657 | 8.265292 | up | COMMD4 |
| 77 | A_23_P91891 | 4.543218 | 2.1837146 | 4.543218 | up | COPB2 |
| 78 | A_33_P3590259 | 15.6784725 | 3.9707131 | 15.6784725 | up | CXCL14 |
| 79 | A_33_P3224045 | 8.20539 | 3.036572 | 8.20539 | up | DAPK3 |
| 80 | A_23_P93311 | 3.6397362 | 1.8638339 | 3.6397362 | up | DDR1 |
| 81 | A_23_P78664 | 9.437213 | 3.238361 | 9.437213 | up | DDX39A |
| 82 | A_33_P3372844 | 18.477966 | 4.207734 | 18.477966 | up | DDX56 |
| 83 | A_24_P215407 | 13.520063 | 3.75703 | 13.520063 | up | DDX6 |
| 84 | A_33_P3239338 | 14.484245 | 3.8564126 | 14.484245 | up | DGKZ |
| 85 | A_23_P24444 | 15.473822 | 3.9517577 | 15.473822 | up | DHCR7 |
| 86 | A_23_P38346 | 7.088444 | 2.825469 | 7.088444 | up | DHX58 |
| 87 | A_23_P47800 | 3.8328323 | 1.9384109 | 3.8328323 | up | DIABLO |
| 88 | A_23_P166899 | 5.8654666 | 2.5522459 | 5.8654666 | up | DNAJB11 |
| 89 | A_24_P225604 | 7.8530197 | 2.9732475 | 7.8530197 | up | DNAJC10 |
| 90 | A_23_P166910 | 6.6366005 | 2.7304444 | 6.6366005 | up | DNAJC13 |
| 91 | A_23_P254573 | 4.42868 | 2.1468768 | 4.42868 | up | DNAJC2 |
| 92 | A_33_P3266898 | 10.132532 | 3.3409228 | 10.132532 | up | DSC3 |
| 93 | A_33_P3402565 | 10.516031 | 3.3945184 | 10.516031 | up | DSP |
| 94 | A_33_P3294392 | 9.21947 | 3.2046838 | 9.21947 | up | EDC4 |
| 95 | A_24_P208045 | 7.767382 | 2.9574285 | 7.767382 | up | EDEM3 |
| 96 | A_23_P113005 | 11.566155 | 3.5318375 | 11.566155 | up | EFNA1 |
| 97 | A_24_P365807 | 97.949066 | 6.61396 | 97.949066 | up | EFNB1 |
| 98 | A_23_P55190 | 13.210368 | 3.7235987 | 13.210368 | up | EFTUD2 |
| 99 | A_23_P142750 | 10.976571 | 3.4563556 | 10.976571 | up | EIF2AK2 |
| 100 | A_23_P157072 | 10.579438 | 3.403191 | 10.579438 | up | EIF3B |
| 101 | A_24_P31235 | 10.4421835 | 3.3843515 | 10.4421835 | up | EIF5A |
| 102 | A_23_P143127 | 5.140595 | 2.3619354 | 5.140595 | up | EML4 |
| 103 | A_23_P168443 | 3.1588252 | 1.6593881 | 3.1588252 | up | EPHB4 |
| 104 | A_23_P100220 | 3.7876105 | 1.921288 | 3.7876105 | up | ESRP2 |
| 105 | A_33_P3233871 | 9.482772 | 3.2453089 | 9.482772 | up | F12 |
| 106 | A_23_P86917 | 5.2526727 | 2.3930516 | 5.2526727 | up | FADD |
| 107 | A_23_P322704 | 10.033113 | 3.3266973 | 10.033113 | up | FAM177A1 |
| 108 | A_33_P3359160 | 5.91218 | 2.5636902 | 5.91218 | up | FAM189B |
| 109 | A_23_P376799 | 3.0699081 | 1.6181955 | 3.0699081 | up | FAM21C |
| 110 | A_23_P163711 | 8.389681 | 3.068616 | 8.389681 | up | FAM57B |
| 111 | A_23_P13663 | 18.310366 | 4.1945887 | 18.310366 | up | FAM60A |
| 112 | A_23_P421175 | 5.32452 | 2.4126515 | 5.32452 | up | FAM83H |
| 113 | A_32_P95729 | 8.065856 | 3.0118277 | 8.065856 | up | FANCI |
| 114 | A_33_P3329344 | 3.184023 | 1.6708508 | 3.184023 | up | FASN |
| 115 | A_24_P93967 | 3.0332687 | 1.6008734 | 3.0332687 | up | FMR1 |
| 116 | A_32_P43050 | 3.0634227 | 1.6151445 | 3.0634227 | up | FRG1 |
| 117 | A_33_P3411075 | 14.869785 | 3.894312 | 14.869785 | up | FSCN1 |
| 118 | A_24_P38276 | 2.5439696 | 1.3470814 | 2.5439696 | up | FZD1 |
| 119 | A_23_P153026 | 9.887375 | 3.3055875 | 9.887375 | up | GAA |
| 120 | A_23_P159775 | 15.218293 | 3.9277346 | 15.218293 | up | GABRE |
| 121 | A_23_P155288 | 3.3669214 | 1.75143 | 3.3669214 | up | GFM1 |
| 122 | A_33_P3252794 | 11.00794 | 3.4604726 | 11.00794 | up | GLI4 |
| 123 | A_23_P78268 | 2.756036 | 1.4625947 | 2.756036 | up | GLOD4 |
| 124 | A_24_P100351 | 5.3950872 | 2.4316463 | 5.3950872 | up | GNL3L |
| 125 | A_23_P429184 | 2.4674835 | 1.3030404 | 2.4674835 | up | GNPNAT1 |
| 126 | A_23_P398275 | 10.188679 | 3.348895 | 10.188679 | up | GOLGA2P7 |
| 127 | A_33_P3414482 | 10.897373 | 3.4459085 | 10.897373 | up | GOT2 |
| 128 | A_33_P3317198 | 3.0565348 | 1.611897 | 3.0565348 | up | GTF2F2 |
| 129 | A_24_P38895 | 12.353457 | 3.626843 | 12.353457 | up | H2AFX |
| 130 | A_23_P103628 | 6.9943895 | 2.8061981 | 6.9943895 | up | HEATR1 |
| 131 | A_33_P3258117 | 4.3356924 | 2.1162624 | 4.3356924 | up | HELLS |
| 132 | A_23_P72627 | 9.120562 | 3.1891227 | 9.120562 | up | HGS |
| 133 | A_23_P122443 | 8.2731495 | 3.0484366 | 8.2731495 | up | HIST1H1C |
| 134 | A_24_P260639 | 7.4178386 | 2.8909988 | 7.4178386 | up | HIST1H1D |
| 135 | A_23_P428184 | 2.8796659 | 1.5259014 | 2.8796659 | up | HIST1H2AD |
| 136 | A_33_P3344086 | 5.337434 | 2.4161463 | 5.337434 | up | HIST1H2AJ |
| 137 | A_24_P217848 | 6.611566 | 2.724992 | 6.611566 | up | HIST1H2AK |
| 138 | A_24_P86389 | 9.596367 | 3.2624884 | 9.596367 | up | HIST1H2AM |
| 139 | A_23_P111054 | 6.2586155 | 2.6458435 | 6.2586155 | up | HIST1H2BB |
| 140 | A_23_P366216 | 4.869422 | 2.2837505 | 4.869422 | up | HIST1H2BH |
| 141 | A_23_P8013 | 5.688141 | 2.5079572 | 5.688141 | up | HIST1H2BL |
| 142 | A_23_P59069 | 5.084401 | 2.346078 | 5.084401 | up | HIST1H2BO |
| 143 | A_23_P93258 | 11.246313 | 3.4913802 | 11.246313 | up | HIST1H3B |
| 144 | A_23_P42198 | 6.279578 | 2.6506677 | 6.279578 | up | HIST1H3G |
| 145 | A_24_P166407 | 6.5582166 | 2.7133036 | 6.5582166 | up | HIST1H4B |
| 146 | A_33_P3410836 | 14.268492 | 3.834761 | 14.268492 | up | HIST1H4D |
| 147 | A_23_P415411 | 9.050609 | 3.1780148 | 9.050609 | up | HIST1H4E |
| 148 | A_23_P359540 | 4.4624043 | 2.1578212 | 4.4624043 | up | HIST1H4F |
| 149 | A_23_P30813 | 24.80083 | 4.6323166 | 24.80083 | up | HIST1H4K |
| 150 | A_24_P68631 | 6.429253 | 2.6846511 | 6.429253 | up | HIST2H2AB |
| 151 | A_23_P301247 | 9.7514105 | 3.285611 | 9.7514105 | up | HIST2H2AC |
| 152 | A_33_P3257678 | 48.71566 | 5.6063137 | 48.71566 | up | HIST2H3A |
| 153 | A_23_P436281 | 11.533105 | 3.527709 | 11.533105 | up | HIST2H4B |
| 154 | A_23_P98431 | 4.808204 | 2.2654982 | 4.808204 | up | HMBS |
| 155 | A_32_P109572 | 8.667727 | 3.1156538 | 8.667727 | up | HNRNPL |
| 156 | A_23_P39364 | 15.998014 | 3.999821 | 15.998014 | up | HOMER3 |
| 157 | A_33_P3377519 | 6.338964 | 2.664247 | 6.338964 | up | HOXA6 |
| 158 | A_23_P2601 | 5.522546 | 2.4653335 | 5.522546 | up | HSP90B1 |
| 159 | A_23_P74843 | 5.961394 | 2.5756497 | 5.961394 | up | IARS2 |
| 160 | A_23_P406135 | 4.994257 | 2.32027 | 4.994257 | up | IFT172 |
| 161 | A_23_P19987 | 11.362252 | 3.506177 | 11.362252 | up | IGF2BP3 |
| 162 | A_23_P85441 | 9.229531 | 3.2062573 | 9.229531 | up | IGSF9 |
| 163 | A_33_P3601163 | 3.4407997 | 1.7827439 | 3.4407997 | up | ILF3-AS1 |
| 164 | A_33_P3316045 | 20.65003 | 4.368072 | 20.65003 | up | INTS1 |
| 165 | A_33_P3321293 | 5.9668093 | 2.5769596 | 5.9668093 | up | IQGAP3 |
| 166 | A_33_P3297562 | 4.549601 | 2.18574 | 4.549601 | up | IRX2 |
| 167 | A_33_P3377364 | 20.107101 | 4.329633 | 20.107101 | up | ITGB4 |
| 168 | A_23_P403424 | 18.358305 | 4.198361 | 18.358305 | up | JMJD7-PLA2G4B |
| 169 | A_23_P66608 | 15.527506 | 3.9567542 | 15.527506 | up | KAT2A |
| 170 | A_33_P3319920 | 5.3994474 | 2.4328117 | 5.3994474 | up | KDM4B |
| 171 | A_23_P119141 | 2.5591636 | 1.3556724 | 2.5591636 | up | KEAP1 |
| 172 | A_23_P16157 | 5.04917 | 2.3360462 | 5.04917 | up | KHSRP |
| 173 | A_23_P106127 | 4.159544 | 2.0564253 | 4.159544 | up | KIAA0586 |
| 174 | A_32_P153725 | 3.2924843 | 1.7191765 | 3.2924843 | up | KIAA1033 |
| 175 | A_23_P215980 | 3.7389555 | 1.9026353 | 3.7389555 | up | KIAA1429 |
| 176 | A_23_P96158 | 10.141595 | 3.3422127 | 10.141595 | up | KRT17 |
| 177 | A_33_P3857239 | 11.545031 | 3.5292 | 11.545031 | up | KRT42P |
| 178 | A_33_P3303810 | 9.588819 | 3.261353 | 9.588819 | up | LAD1 |
| 179 | A_33_P3338121 | 8.118934 | 3.0212903 | 8.118934 | up | LAMB3 |
| 180 | A_33_P3246068 | 4.462661 | 2.1579041 | 4.462661 | up | LAMTOR2 |
| 181 | A_23_P375524 | 4.906631 | 2.2947328 | 4.906631 | up | LCE1D |
| 182 | A_33_P3619819 | 5.383109 | 2.4284396 | 5.383109 | up | lnc-USP35-1 |
| 183 | A_33_P3280094 | 31.449976 | 4.974987 | 31.449976 | up | LRFN4 |
| 184 | A_33_P3221064 | 3.0927296 | 1.6288807 | 3.0927296 | up | LTBP4 |
| 185 | A_33_P3236651 | 9.115673 | 3.1883492 | 9.115673 | up | MAP2K7 |
| 186 | A_33_P3301851 | 8.812574 | 3.1395636 | 8.812574 | up | MAP3K11 |
| 187 | A_23_P70047 | 13.636955 | 3.7694497 | 13.636955 | up | MATR3 |
| 188 | A_33_P3352767 | 6.500085 | 2.7004585 | 6.500085 | up | MC1R |
| 189 | A_33_P3339036 | 5.7636814 | 2.5269907 | 5.7636814 | up | MECP2 |
| 190 | A_33_P3278293 | 3.1453938 | 1.6532407 | 3.1453938 | up | METTL16 |
| 191 | A_33_P3343785 | 3.3467774 | 1.7427726 | 3.3467774 | up | MGME1 |
| 192 | A_24_P303524 | 13.054293 | 3.7064524 | 13.054293 | up | MICALL2 |
| 193 | A_23_P158997 | 4.3262086 | 2.1131032 | 4.3262086 | up | MIR3916 |
| 194 | A_33_P3374210 | 5.3606596 | 2.4224105 | 5.3606596 | up | MKI67 |
| 195 | A_24_P82106 | 10.27472 | 3.3610272 | 10.27472 | up | MMP14 |
| 196 | A_33_P3390477 | 10.130212 | 3.3405924 | 10.130212 | up | MRPL4 |
| 197 | A_23_P258321 | 12.63548 | 3.6594086 | 12.63548 | up | MRPS17 |
| 198 | A_23_P59107 | 3.8284185 | 1.9367485 | 3.8284185 | up | MRPS18B |
| 199 | A_33_P3333527 | 5.582607 | 2.480939 | 5.582607 | up | MSTO1 |
| 200 | A_33_P3297255 | 2.2916014 | 1.1963562 | 2.2916014 | up | MXD3 |
| 201 | A_23_P78170 | 3.5273569 | 1.8185875 | 3.5273569 | up | MYBBP1A |
| 202 | A_23_P320250 | 6.2776327 | 2.6502206 | 6.2776327 | up | MYO19 |
| 203 | A_24_P255218 | 10.090459 | 3.33492 | 10.090459 | up | MYO5A |
| 204 | A_33_P3248794 | 14.151388 | 3.8228717 | 14.151388 | up | NAB2 |
| 205 | A_33_P3275330 | 6.1936536 | 2.6307907 | 6.1936536 | up | NADK |
| 206 | A_33_P3272558 | 10.405276 | 3.3792434 | 10.405276 | up | NCAPH2 |
| 207 | A_23_P34402 | 7.252856 | 2.858549 | 7.252856 | up | NCSTN |
| 208 | A_33_P3317406 | 9.738764 | 3.2837386 | 9.738764 | up | NDUFV1 |
| 209 | A_24_P219552 | 16.302032 | 4.02698 | 16.302032 | up | NFE2L1 |
| 210 | A_33_P3283971 | 2.004403 | 1.0031726 | 2.004403 | up | NFKBIL1 |
| 211 | A_23_P140602 | 2.8020868 | 1.4865017 | 2.8020868 | up | NGRN |
| 212 | A_23_P32861 | 3.4668443 | 1.7936231 | 3.4668443 | up | NMD3 |
| 213 | A_23_P152115 | 9.22356 | 3.2053237 | 9.22356 | up | NME3 |
| 214 | A_24_P14010 | 12.241571 | 3.6137168 | 12.241571 | up | NMT1 |
| 215 | A_23_P206371 | 7.008612 | 2.8091288 | 7.008612 | up | NOL3 |
| 216 | A_33_P3389638 | 2.7609522 | 1.465166 | 2.7609522 | up | NOP14 |
| 217 | A_33_P3313055 | 25.288038 | 4.660383 | 25.288038 | up | NOTCH3 |
| 218 | A_24_P45005 | 5.707346 | 2.51282 | 5.707346 | up | NPEPL1 |
| 219 | A_33_P3238966 | 7.1904817 | 2.8460884 | 7.1904817 | up | NPLOC4 |
| 220 | A_23_P100326 | 7.5114007 | 2.909082 | 7.5114007 | up | NPRL3 |
| 221 | A_23_P82412 | 5.1143413 | 2.3545485 | 5.1143413 | up | NSUN5 |
| 222 | A_23_P14062 | 3.3686116 | 1.7521541 | 3.3686116 | up | NUP107 |
| 223 | A_32_P51084 | 11.798672 | 3.5605526 | 11.798672 | up | NUP205 |
| 224 | A_33_P3320548 | 5.349719 | 2.4194632 | 5.349719 | up | NUPL2 |
| 225 | A_23_P96990 | 5.3195543 | 2.4113054 | 5.3195543 | up | NVL |
| 226 | A_33_P3417695 | 7.877412 | 2.9777217 | 7.877412 | up | ODF3B |
| 227 | A_33_P3245927 | 6.0472198 | 2.596272 | 6.0472198 | up | OGFOD2 |
| 228 | A_23_P138137 | 3.5149274 | 1.8134949 | 3.5149274 | up | OMA1 |
| 229 | A_23_P170186 | 6.475956 | 2.6950932 | 6.475956 | up | OPLAH |
| 230 | A_33_P3362088 | 9.623033 | 3.2664917 | 9.623033 | up | P2RX4 |
| 231 | A_33_P3257030 | 32.209167 | 5.0093994 | 32.209167 | up | P3H4 |
| 232 | A_33_P3227209 | 5.3773217 | 2.4268878 | 5.3773217 | up | PA2G4 |
| 233 | A_33_P3413808 | 3.3922591 | 1.7622464 | 3.3922591 | up | PABPC1L |
| 234 | A_33_P3398448 | 22.766153 | 4.5088186 | 22.766153 | up | PARP10 |
| 235 | A_23_P53663 | 9.582672 | 3.260428 | 9.582672 | up | PAWR |
| 236 | A_23_P252681 | 6.4878902 | 2.6977494 | 6.4878902 | up | PCYT1A |
| 237 | A_24_P101201 | 14.685557 | 3.876326 | 14.685557 | up | PDIA3 |
| 238 | A_33_P3254634 | 3.6383445 | 1.8632822 | 3.6383445 | up | PDIA5 |
| 239 | A_33_P3369761 | 7.8954363 | 2.981019 | 7.8954363 | up | PDP1 |
| 240 | A_33_P3659808 | 6.9715185 | 2.801473 | 6.9715185 | up | PELP1 |
| 241 | A_33_P3398697 | 7.613419 | 2.9285445 | 7.613419 | up | PICK1 |
| 242 | A_33_P3812815 | 12.480538 | 3.6416082 | 12.480538 | up | PKD1 |
| 243 | A_23_P399501 | 17.897533 | 4.161689 | 17.897533 | up | PKM |
| 244 | A_33_P3397443 | 4.969271 | 2.3130343 | 4.969271 | up | PKMYT1 |
| 245 | A_33_P3224745 | 6.148695 | 2.6202803 | 6.148695 | up | PLA2G15 |
| 246 | A_33_P3306146 | 34.530376 | 5.109794 | 34.530376 | up | PLAU |
| 247 | A_33_P3334180 | 7.4428096 | 2.8958473 | 7.4428096 | up | PLCH2 |
| 248 | A_32_P57728 | 5.971455 | 2.5780826 | 5.971455 | up | PMS2P1 |
| 249 | A_23_P154488 | 4.8432345 | 2.275971 | 4.8432345 | up | PNPT1 |
| 250 | A_23_P50455 | 3.6732063 | 1.8770399 | 3.6732063 | up | POLD1 |
| 251 | A_33_P3326423 | 3.6459093 | 1.8662786 | 3.6459093 | up | POLL |
| 252 | A_33_P3218138 | 8.689909 | 3.1193411 | 8.689909 | up | POLR2A |
| 253 | A_33_P3284197 | 8.252163 | 3.0447724 | 8.252163 | up | POMGNT1 |
| 254 | A_24_P237804 | 7.9536667 | 2.99162 | 7.9536667 | up | POTED |
| 255 | A_24_P825874 | 13.8393545 | 3.7907047 | 13.8393545 | up | POTEI |
| 256 | A_23_P302116 | 5.56845 | 2.4772758 | 5.56845 | up | PPFIA1 |
| 257 | A_23_P157715 | 11.444312 | 3.516559 | 11.444312 | up | PPP1R16A |
| 258 | A_24_P376309 | 7.8455467 | 2.971874 | 7.8455467 | up | PPP1R9B |
| 259 | A_24_P294931 | 6.940904 | 2.7951236 | 6.940904 | up | PPP2R5D |
| 260 | A_33_P3445805 | 7.4157043 | 2.8905838 | 7.4157043 | up | PPP6R3 |
| 261 | A_24_P396197 | 5.0058956 | 2.3236282 | 5.0058956 | up | PRKCSH |
| 262 | A_23_P77430 | 3.6658094 | 1.8741318 | 3.6658094 | up | PRMT7 |
| 263 | A_33_P3363012 | 14.255267 | 3.8334231 | 14.255267 | up | PRPF19 |
| 264 | A_23_P170058 | 3.604978 | 1.8499905 | 3.604978 | up | PSMB2 |
| 265 | A_33_P3234899 | 6.218212 | 2.6365 | 6.218212 | up | PSMB3 |
| 266 | A_23_P75889 | 2.8427765 | 1.5073007 | 2.8427765 | up | PSMD13 |
| 267 | A_23_P434301 | 2.9433944 | 1.5574809 | 2.9433944 | up | PTMA |
| 268 | A_24_P2648 | 6.701934 | 2.7445774 | 6.701934 | up | PTPN14 |
| 269 | A_33_P3296497 | 4.831952 | 2.2726061 | 4.831952 | up | PTPRK |
| 270 | A_24_P149645 | 4.137311 | 2.0486934 | 4.137311 | up | PUF60 |
| 271 | A_23_P65466 | 4.989971 | 2.3190315 | 4.989971 | up | RAB2B |
| 272 | A_33_P3320127 | 4.3633676 | 2.125442 | 4.3633676 | up | RAB8A |
| 273 | A_33_P3424207 | 9.990948 | 3.3206215 | 9.990948 | up | RABEP2 |
| 274 | A_33_P3411991 | 6.860565 | 2.7783275 | 6.860565 | up | RABGAP1 |
| 275 | A_33_P3295523 | 9.603496 | 3.2635596 | 9.603496 | up | RAC3 |
| 276 | A_23_P74115 | 3.1018767 | 1.6331414 | 3.1018767 | up | RAD54L |
| 277 | A_33_P3443165 | 13.647024 | 3.7705145 | 13.647024 | up | RAE1 |
| 278 | A_23_P310331 | 4.072373 | 2.0258696 | 4.072373 | up | RANBP3 |
| 279 | A_33_P3263307 | 14.550854 | 3.8630319 | 14.550854 | up | RANGAP1 |
| 280 | A_32_P5251 | 4.6048145 | 2.2031431 | 4.6048145 | up | RARA |
| 281 | A_33_P3283420 | 3.7103481 | 1.8915546 | 3.7103481 | up | RBM12B |
| 282 | A_23_P150255 | 3.4990458 | 1.8069615 | 3.4990458 | up | RBM14 |
| 283 | A_33_P3323847 | 14.519592 | 3.859929 | 14.519592 | up | RECQL4 |
| 284 | A_33_P3272160 | 6.965461 | 2.8002188 | 6.965461 | up | REXO4 |
| 285 | A_23_P8561 | 3.009128 | 1.5893456 | 3.009128 | up | RHBDD2 |
| 286 | A_33_P3274069 | 11.301721 | 3.4984705 | 11.301721 | up | RHBDD3 |
| 287 | A_23_P314086 | 8.122031 | 3.0218406 | 8.122031 | up | RNF126 |
| 288 | A_33_P3329878 | 3.7593462 | 1.9104818 | 3.7593462 | up | RNF216 |
| 289 | A_23_P64630 | 5.149118 | 2.3643253 | 5.149118 | up | RNF26 |
| 290 | A_23_P29594 | 5.9754205 | 2.5790403 | 5.9754205 | up | RPL39L |
| 291 | A_33_P3382513 | 10.667211 | 3.415111 | 10.667211 | up | RPUSD1 |
| 292 | A_23_P120566 | 6.495124 | 2.699357 | 6.495124 | up | RRBP1 |
| 293 | A_23_P395534 | 5.995303 | 2.5838327 | 5.995303 | up | RSC1A1 |
| 294 | A_33_P3211804 | 11.348925 | 3.5044837 | 11.348925 | up | RUNX1 |
| 295 | A_33_P3346791 | 11.683503 | 3.546401 | 11.683503 | up | SCAMP3 |
| 296 | A_33_P3290443 | 7.9439025 | 2.989848 | 7.9439025 | up | SCARNA9 |
| 297 | A_23_P159039 | 2.894285 | 1.5332069 | 2.894285 | up | SCRIB |
| 298 | A_23_P6344 | 9.158721 | 3.195146 | 9.158721 | up | SDF2L1 |
| 299 | A_33_P3290672 | 6.216706 | 2.6361504 | 6.216706 | up | SELT |
| 300 | A_24_P228637 | 3.8418396 | 1.9417973 | 3.8418396 | up | SETD4 |
| 301 | A_23_P80278 | 4.4387555 | 2.1501553 | 4.4387555 | up | SFI1 |
| 302 | A_33_P3389286 | 49.92126 | 5.6415825 | 49.92126 | up | SFN |
| 303 | A_33_P3213551 | 7.159973 | 2.8399541 | 7.159973 | up | SHARPIN |
| 304 | A_33_P3229181 | 14.054225 | 3.812932 | 14.054225 | up | SLC12A9 |
| 305 | A_33_P3245066 | 2.27037 | 1.1829275 | 2.27037 | up | SLC35E2B |
| 306 | A_23_P55011 | 9.036798 | 3.1758115 | 9.036798 | up | SLC38A10 |
| 307 | A_33_P3240353 | 9.848681 | 3.2999306 | 9.848681 | up | SLC39A4 |
| 308 | A_23_P75811 | 3.70352 | 1.8888972 | 3.70352 | up | SLC3A2 |
| 309 | A_23_P154688 | 13.095058 | 3.7109506 | 13.095058 | up | SLC4A11 |
| 310 | A_33_P3274696 | 16.657255 | 4.058079 | 16.657255 | up | SLC52A2 |
| 311 | A_33_P3402615 | 5.049108 | 2.3360286 | 5.049108 | up | SLC6A9 |
| 312 | A_23_P333063 | 2.0567727 | 1.0403824 | 2.0567727 | up | SMARCE1 |
| 313 | A_33_P3326349 | 5.373957 | 2.4259849 | 5.373957 | up | SMG5 |
| 314 | A_23_P255286 | 17.034626 | 4.0903983 | 17.034626 | up | SMPD4 |
| 315 | A_23_P393607 | 10.665659 | 3.4149013 | 10.665659 | up | SNAP47 |
| 316 | A_23_P20722 | 17.75934 | 4.150506 | 17.75934 | up | SNAPC4 |
| 317 | A_33_P3280965 | 2.2709997 | 1.1833274 | 2.2709997 | up | SNHG9 |
| 318 | A_33_P3578325 | 24.183754 | 4.5959663 | 24.183754 | up | SNORD15A |
| 319 | A_33_P3263379 | 34.786537 | 5.120457 | 34.786537 | up | SNORD17 |
| 320 | A_33_P3226542 | 12.448795 | 3.6379342 | 12.448795 | up | SNORD3B-1 |
| 321 | A_23_P154675 | 5.7760715 | 2.5300887 | 5.7760715 | up | SNRPB |
| 322 | A_23_P7313 | 46.787704 | 5.5480576 | 46.787704 | up | SPP1 |
| 323 | A_23_P337201 | 5.3062606 | 2.4076955 | 5.3062606 | up | SRP72 |
| 324 | A_23_P154025 | 7.225932 | 2.8531837 | 7.225932 | up | SSB |
| 325 | A_23_P68910 | 17.15642 | 4.1006765 | 17.15642 | up | SSTR3 |
| 326 | A_33_P3213064 | 16.196766 | 4.017634 | 16.196766 | up | STAT2 |
| 327 | A_33_P3389148 | 28.807346 | 4.848365 | 28.807346 | up | STK11 |
| 328 | A_23_P321320 | 4.491372 | 2.1671562 | 4.491372 | up | STK11IP |
| 329 | A_23_P54376 | 9.019793 | 3.1730943 | 9.019793 | up | STOML1 |
| 330 | A_23_P154605 | 18.306286 | 4.1942673 | 18.306286 | up | SULF2 |
| 331 | A_32_P192615 | 4.3403487 | 2.117811 | 4.3403487 | up | TAF1 |
| 332 | A_23_P74663 | 3.2600172 | 1.7048795 | 3.2600172 | up | TAF1A |
| 333 | A_33_P3348614 | 5.7675242 | 2.5279522 | 5.7675242 | up | TBL1XR1 |
| 334 | A_33_P3219965 | 21.30472 | 4.413101 | 21.30472 | up | TCIRG1 |
| 335 | A_23_P65481 | 4.2599626 | 2.0908408 | 4.2599626 | up | TEP1 |
| 336 | A_23_P45940 | 3.433111 | 1.7795165 | 3.433111 | up | TFB2M |
| 337 | A_23_P209933 | 6.331047 | 2.662444 | 6.331047 | up | TGOLN2 |
| 338 | A_33_P3336642 | 6.86613 | 2.7794971 | 6.86613 | up | THOC3 |
| 339 | A_23_P107421 | 19.10264 | 4.2557 | 19.10264 | up | TK1 |
| 340 | A_23_P38365 | 8.785232 | 3.1350803 | 8.785232 | up | TLK2 |
| 341 | A_33_P3320368 | 5.9990954 | 2.584745 | 5.9990954 | up | TMEM161A |
| 342 | A_33_P3422248 | 104.38099 | 6.705715 | 104.38099 | up | TMEM200C |
| 343 | A_23_P206369 | 6.9262695 | 2.7920785 | 6.9262695 | up | TMEM208 |
| 344 | A_33_P3380982 | 2.2661128 | 1.1802197 | 2.2661128 | up | TMEM30A |
| 345 | A_24_P307289 | 11.971709 | 3.5815573 | 11.971709 | up | TMEM95 |
| 346 | A_23_P126844 | 14.276915 | 3.8356123 | 14.276915 | up | TNFRSF25 |
| 347 | A_33_P3423300 | 15.008746 | 3.9077315 | 15.008746 | up | TNPO2 |
| 348 | A_23_P207850 | 27.46244 | 4.779388 | 27.46244 | up | TNS4 |
| 349 | A_33_P3274199 | 11.765568 | 3.556499 | 11.765568 | up | TP53I13 |
| 350 | A_23_P99424 | 3.178382 | 1.6682925 | 3.178382 | up | TPP2 |
| 351 | A_24_P179611 | 9.532743 | 3.2528915 | 9.532743 | up | TPR |
| 352 | A_33_P3803639 | 5.7208323 | 2.516225 | 5.7208323 | up | TRAF2 |
| 353 | A_23_P425880 | 5.5957828 | 2.48434 | 5.5957828 | up | TRIO |
| 354 | A_33_P3238976 | 5.5649104 | 2.4763584 | 5.5649104 | up | TRMT5 |
| 355 | A_33_P3312258 | 3.8362088 | 1.9396813 | 3.8362088 | up | TUBB |
| 356 | A_24_P297539 | 16.154472 | 4.0138617 | 16.154472 | up | UBE2C |
| 357 | A_24_P329600 | 5.5102186 | 2.4621096 | 5.5102186 | up | UBQLN1 |
| 358 | A_23_P362637 | 4.223936 | 2.078588 | 4.223936 | up | UBR2 |
| 359 | A_33_P3366246 | 4.7528987 | 2.2488077 | 4.7528987 | up | UGGT1 |
| 360 | A_23_P342067 | 2.2979784 | 1.2003653 | 2.2979784 | up | UHMK1 |
| 361 | A_33_P3403748 | 5.2518272 | 2.3928194 | 5.2518272 | up | UQCC3 |
| 362 | A_33_P3316026 | 7.709605 | 2.946657 | 7.709605 | up | USF2 |
| 363 | A_23_P100196 | 3.3211486 | 1.7316823 | 3.3211486 | up | USP10 |
| 364 | A_23_P164341 | 15.763042 | 3.9784741 | 15.763042 | up | VAMP2 |
| 365 | A_33_P3248664 | 6.936512 | 2.7942104 | 6.936512 | up | VARS |
| 366 | A_23_P111112 | 9.462159 | 3.2421694 | 9.462159 | up | VARS2 |
| 367 | A_23_P119102 | 11.299203 | 3.4981492 | 11.299203 | up | VASP |
| 368 | A_23_P83045 | 11.402642 | 3.5112963 | 11.402642 | up | VCP |
| 369 | A_23_P11744 | 11.047067 | 3.4655914 | 11.047067 | up | WASH1 |
| 370 | A_33_P3411925 | 6.894024 | 2.7853463 | 6.894024 | up | WDR18 |
| 371 | A_33_P3248759 | 11.555599 | 3.5305202 | 11.555599 | up | WDR24 |
| 372 | A_23_P110076 | 2.6102617 | 1.3841945 | 2.6102617 | up | WDR53 |
| 373 | A_33_P3510335 | 3.1022348 | 1.6333079 | 3.1022348 | up | WNK1 |
| 374 | A_33_P3292130 | 8.684317 | 3.1184123 | 8.684317 | up | XLOC_l2_013383 |
| 375 | A_23_P25224 | 6.1666384 | 2.6244843 | 6.1666384 | up | YBX3 |
| 376 | A_23_P5912 | 7.6206007 | 2.9299047 | 7.6206007 | up | YTHDF1 |
| 377 | A_23_P82108 | 3.4247022 | 1.7759786 | 3.4247022 | up | ZBTB2 |
| 378 | A_33_P3273969 | 9.272299 | 3.212927 | 9.272299 | up | ZC3H11A |
| 379 | A_33_P3423820 | 13.4298935 | 3.747376 | 13.4298935 | up | ZC3H3 |
| 380 | A_33_P3356701 | 7.605888 | 2.9271166 | 7.605888 | up | ZDHHC12 |
| 381 | A_23_P323924 | 9.834516 | 3.297854 | 9.834516 | up | ZFYVE27 |
| 382 | A_33_P3234939 | 3.1537058 | 1.6570481 | 3.1537058 | up | ZNF266 |
| 383 | A_33_P3394183 | 3.641951 | 1.8647115 | 3.641951 | up | ZNF324B |
| 384 | A_33_P3257528 | 2.4152865 | 1.2721944 | 2.4152865 | up | ZNF451 |
| 385 | A_23_P28015 | 3.0022836 | 1.5860603 | 3.0022836 | up | ZNF558 |
| 386 | A_33_P3403778 | 7.7479725 | 2.9538188 | 7.7479725 | up | ZNF579 |
| 387 | A_33_P3290487 | 6.341794 | 2.664891 | 6.341794 | up | ZNF668 |
