# Supplementary material for: The miR-1224-5p/TNS4/EGFR axis inhibits tumour progression in oesophageal squamous cell carcinoma
Source: Cell Death Dis. 2020 Jul 30;11(7):597. doi: 10.1038/s41419-020-02801-6 (PMC7393493; doi:10.1038/s41419-020-02801-6)
Supplement: Supplementary file 4 — Table S4 [file 41419_2020_2801_MOESM4_ESM.docx]

**Table S4. Gene ontology of differentially expressed genes in ESCC**

| NO. | GO accession | GO term | *p* value | Corrected *p* value |
| --- | --- | --- | --- | --- |
| 1 | GO:0000786\|GO:0005718 | nucleosome | 2.05E-14 | 1.07E-09 |
| 2 | GO:0044815 | DNA packaging complex | 7.40E-14 | 1.92E-09 |
| 3 | GO:0044446 | intracellular organelle part | 1.89E-13 | 3.28E-09 |
| 4 | GO:0043233 | organelle lumen | 8.22E-13 | 5.35E-09 |
| 5 | GO:0070013 | intracellular organelle lumen | 8.22E-13 | 5.35E-09 |
| 6 | GO:0043227 | membrane-bounded organelle | 7.81E-13 | 5.35E-09 |
| 7 | GO:0031974 | membrane-enclosed lumen | 8.22E-13 | 5.35E-09 |
| 8 | GO:0044422 | organelle part | 5.46E-13 | 5.35E-09 |
| 9 | GO:0032993 | protein-DNA complex | 1.01E-12 | 5.84E-09 |
| 10 | GO:0043226 | organelle | 1.01E-11 | 5.27E-08 |
| 11 | GO:0044428 | nuclear part | 5.66E-10 | 2.68E-06 |
| 12 | GO:0043231 | intracellular membrane-bounded organelle | 6.63E-10 | 2.88E-06 |
| 13 | GO:0031981 | nuclear lumen | 7.71E-10 | 3.09E-06 |
| 14 | GO:0006342\|GO:0016440 | chromatin silencing | 9.09E-10 | 3.38E-06 |
| 15 | GO:0000785\|GO:0005717 | chromatin | 1.48E-09 | 5.14E-06 |
| 16 | GO:0044424 | intracellular part | 1.81E-09 | 5.24E-06 |
| 17 | GO:0006333 | chromatin assembly or disassembly | 1.79E-09 | 5.24E-06 |
| 18 | GO:0006323 | DNA packaging | 1.77E-09 | 5.24E-06 |
| 19 | GO:0044427 | chromosomal part | 2.86E-09 | 7.82E-06 |
| 20 | GO:0065004 | protein-DNA complex assembly | 3.49E-09 | 9.09E-06 |
| 21 | GO:0071103 | DNA conformation change | 4.99E-09 | 1.24E-05 |
| 22 | GO:0031497 | chromatin assembly | 6.78E-09 | 1.60E-05 |
| 23 | GO:0071824 | protein-DNA complex subunit organization | 8.25E-09 | 1.87E-05 |
| 24 | GO:0005622 | intracellular | 1.00E-08 | 1.89E-05 |
| 25 | GO:1903561 | extracellular vesicle | 9.48E-09 | 1.89E-05 |
| 26 | GO:0006334 | nucleosome assembly | 1.02E-08 | 1.89E-05 |
| 27 | GO:0043230 | extracellular organelle | 9.68E-09 | 1.89E-05 |
| 28 | GO:0070062 | extracellular exosome | 9.48E-09 | 1.89E-05 |
| 29 | GO:0000183 | chromatin silencing at rDNA | 1.67E-08 | 2.92E-05 |
| 30 | GO:0005654 | nucleoplasm | 1.69E-08 | 2.92E-05 |
| 31 | GO:0005515\|GO:0001948\|GO:0045308 | protein binding | 1.82E-08 | 3.06E-05 |
| 32 | GO:0071840\|GO:0071841 | cellular component organization or biogenesis | 1.98E-08 | 3.22E-05 |
| 33 | GO:0043229 | intracellular organelle | 2.15E-08 | 3.39E-05 |
| 34 | GO:0034728 | nucleosome organization | 2.67E-08 | 4.09E-05 |
| 35 | GO:0006335 | DNA replication-dependent nucleosome assembly | 4.42E-08 | 6.39E-05 |
| 36 | GO:0034723 | DNA replication-dependent nucleosome organization | 4.42E-08 | 6.39E-05 |
| 37 | GO:0005694 | chromosome | 4.80E-08 | 6.75E-05 |
| 38 | GO:0034622\|GO:0043623 | cellular protein-containing complex assembly | 5.03E-08 | 6.89E-05 |
| 39 | GO:0032776 | DNA methylation on cytosine | 8.08E-08 | 1.05E-04 |
| 40 | GO:0005634 | nucleus | 7.91E-08 | 1.05E-04 |
| 41 | GO:0043170\|GO:0043283\|GO:0044259 | macromolecule metabolic process | 1.10E-07 | 1.40E-04 |
| 42 | GO:0050658 | RNA transport | 1.52E-07 | 1.79E-04 |
| 43 | GO:0050657 | nucleic acid transport | 1.52E-07 | 1.79E-04 |
| 44 | GO:0051236 | establishment of RNA localization | 1.52E-07 | 1.79E-04 |
| 45 | GO:0043232 | intracellular non-membrane-bounded organelle | 2.89E-07 | 3.27E-04 |
| 46 | GO:0043228 | non-membrane-bounded organelle | 2.89E-07 | 3.27E-04 |
| 47 | GO:0006403 | RNA localization | 3.61E-07 | 4.00E-04 |
| 48 | GO:0051028 | mRNA transport | 4.47E-07 | 4.85E-04 |
| 49 | GO:0016043\|GO:0044235\|GO:0071842 | cellular component organization | 4.60E-07 | 4.88E-04 |
| 50 | GO:0031982\|GO:0031988 | vesicle | 5.56E-07 | 5.79E-04 |
| 51 | GO:0051641\|GO:1902580 | cellular localization | 7.69E-07 | 7.85E-04 |
| 52 | GO:0006306 | DNA methylation | 9.11E-07 | 8.94E-04 |
| 53 | GO:0006305 | DNA alkylation | 9.11E-07 | 8.94E-04 |
| 54 | GO:0035575 | histone demethylase activity (H4-K20 specific) | 9.86E-07 | 9.16E-04 |
| 55 | GO:0035574 | histone H4-K20 demethylation | 9.86E-07 | 9.16E-04 |
| 56 | GO:0010467 | gene expression | 9.57E-07 | 9.16E-04 |
| 57 | GO:0006996\|GO:1902589 | organelle organization | 1.03E-06 | 9.43E-04 |
| 58 | GO:0032991\|GO:0043234 | protein-containing complex | 1.33E-06 | 0.001193551 |
| 59 | GO:0005737 | cytoplasm | 1.50E-06 | 0.001325575 |
| 60 | GO:0015931 | nucleobase-containing compound transport | 1.85E-06 | 0.001604901 |
| 61 | GO:0016458 | gene silencing | 2.26E-06 | 0.001931595 |
| 62 | GO:0045653 | negative regulation of megakaryocyte differentiation | 2.34E-06 | 0.00196021 |
| 63 | GO:0045814 | negative regulation of gene expression, epigenetic | 2.46E-06 | 0.00202859 |
| 64 | GO:0005829 | cytosol | 2.60E-06 | 0.002116281 |
| 65 | GO:0044085\|GO:0071843 | cellular component biogenesis | 3.47E-06 | 0.002776421 |
| 66 | GO:0006807 | nitrogen compound metabolic process | 3.58E-06 | 0.002823622 |
| 67 | GO:0006325\|GO:0016568 | chromatin organization | 3.86E-06 | 0.002999602 |
| 68 | GO:0034641 | cellular nitrogen compound metabolic process | 4.37E-06 | 0.00326606 |
| 69 | GO:0051649 | establishment of localization in cell | 4.39E-06 | 0.00326606 |
| 70 | GO:0044728 | DNA methylation or demethylation | 6.85E-06 | 0.005017528 |
| 71 | GO:0005615 | extracellular space | 7.15E-06 | 0.005170408 |
| 72 | GO:0007049 | cell cycle | 8.00E-06 | 0.005701429 |
| 73 | GO:0006611\|GO:0097349 | protein export from nucleus | 8.34E-06 | 0.005864451 |
| 74 | GO:0044421 | extracellular region part | 9.67E-06 | 0.006682631 |
| 75 | GO:0031055 | chromatin remodeling at centromere | 9.76E-06 | 0.006682631 |
| 76 | GO:0019899 | enzyme binding | 1.57E-05 | 0.010586636 |
| 77 | GO:0065003\|GO:0006461 | protein-containing complex assembly | 1.62E-05 | 0.010816221 |
| 78 | GO:0044238 | primary metabolic process | 1.81E-05 | 0.011922782 |
| 79 | GO:0034508 | centromere complex assembly | 1.94E-05 | 0.012611846 |
| 80 | GO:0051168 | nuclear export | 2.01E-05 | 0.012906332 |
| 81 | GO:0051276\|GO:0007001\|GO:0051277 | chromosome organization | 2.19E-05 | 0.013906608 |
| 82 | GO:0046907\|GO:1902582 | intracellular transport | 2.42E-05 | 0.01519263 |
| 83 | GO:0051292 | nuclear pore complex assembly | 2.51E-05 | 0.015539372 |
| 84 | GO:0071166 | ribonucleoprotein complex localization | 2.70E-05 | 0.016328823 |
| 85 | GO:0071426 | ribonucleoprotein complex export from nucleus | 2.70E-05 | 0.016328823 |
| 86 | GO:0005488 | binding | 3.22E-05 | 0.019275859 |
| 87 | GO:0044444 | cytoplasmic part | 3.33E-05 | 0.019700328 |
| 88 | GO:0006405 | RNA export from nucleus | 3.75E-05 | 0.02143437 |
| 89 | GO:0006304 | DNA modification | 3.73E-05 | 0.02143437 |
| 90 | GO:0043933\|GO:0034600\|GO:0034621\|GO:0071822 | protein-containing complex subunit organization | 3.67E-05 | 0.02143437 |
| 91 | GO:0071705 | nitrogen compound transport | 3.81E-05 | 0.021546176 |
| 92 | GO:0006259\|GO:0055132 | DNA metabolic process | 3.89E-05 | 0.021763526 |
| 93 | GO:0019538\|GO:0006411\|GO:0044268 | protein metabolic process | 3.96E-05 | 0.021909323 |
| 94 | GO:0071203 | WASH complex | 4.43E-05 | 0.02424256 |
| 95 | GO:0045652 | regulation of megakaryocyte differentiation | 4.52E-05 | 0.024477666 |
| 96 | GO:0071427 | mRNA-containing ribonucleoprotein complex export from nucleus | 6.00E-05 | 0.031565476 |
| 97 | GO:0006406 | mRNA export from nucleus | 6.00E-05 | 0.031565476 |
| 98 | GO:0061641 | CENP-A containing chromatin organization | 6.34E-05 | 0.0326775 |
| 99 | GO:0034080\|GO:0034509 | CENP-A containing nucleosome assembly | 6.34E-05 | 0.0326775 |
| 100 | GO:0071704 | organic substance metabolic process | 6.45E-05 | 0.03292661 |
| 101 | GO:0043044 | ATP-dependent chromatin remodeling | 6.73E-05 | 0.033985697 |
| 102 | GO:0044267 | cellular protein metabolic process | 7.07E-05 | 0.035400804 |
| 103 | GO:0006999 | nuclear pore organization | 7.23E-05 | 0.035833195 |
| 104 | GO:0033036 | macromolecule localization | 7.37E-05 | 0.03620109 |
| 105 | GO:0007077 | mitotic nuclear envelope disassembly | 7.45E-05 | 0.036224324 |
| 106 | GO:0022607\|GO:0071844 | cellular component assembly | 7.77E-05 | 0.037443683 |
| 107 | GO:0003723\|GO:0044822 | RNA binding | 8.64E-05 | 0.041140843 |
| 108 | GO:0046982 | protein heterodimerization activity | 8.97E-05 | 0.042041358 |
| 109 | GO:0008104\|GO:0008105 | protein localization | 9.46E-05 | 0.043935463 |
| 110 | GO:0051179\|GO:1902578 | localization | 9.95E-05 | 0.04584452 |
| 111 | GO:0030397 | membrane disassembly | 1.01E-04 | 0.045854118 |
| 112 | GO:0051081 | nuclear envelope disassembly | 1.01E-04 | 0.045854118 |
| 113 | GO:0046825 | regulation of protein export from nucleus | 1.03E-04 | 0.046289653 |
| 114 | GO:0005623 | cell | 1.06E-04 | 0.04674656 |
| 115 | GO:0044464 | cell part | 1.06E-04 | 0.04674656 |
| 116 | GO:0033157 | regulation of intracellular protein transport | 1.07E-04 | 0.04687987 |
| 117 | GO:1901564 | organonitrogen compound metabolic process | 1.20E-04 | 0.052086134 |
| 118 | GO:0031503 | protein-containing complex localization | 1.31E-04 | 0.05617612 |
| 119 | GO:0010498 | proteasomal protein catabolic process | 1.45E-04 | 0.061747335 |
| 120 | GO:0032452 | histone demethylase activity | 1.49E-04 | 0.062946364 |
| 121 | GO:0000278\|GO:0007067 | mitotic cell cycle | 1.59E-04 | 0.06691452 |
| 122 | GO:0046931 | pore complex assembly | 1.64E-04 | 0.06817424 |
| 123 | GO:0071702 | organic substance transport | 1.73E-04 | 0.071584634 |
| 124 | GO:0070076 | histone lysine demethylation | 1.77E-04 | 0.07247743 |
| 125 | GO:0000228 | nuclear chromosome | 1.80E-04 | 0.07324197 |
| 126 | GO:0008152\|GO:0044236\|GO:0044710 | metabolic process | 2.08E-04 | 0.08391618 |
| 127 | GO:0005643\|GO:0005644 | nuclear pore | 2.10E-04 | 0.0840796 |
| 128 | GO:0006913\|GO:0000063 | nucleocytoplasmic transport | 2.12E-04 | 0.08419473 |
| 129 | GO:0051169 | nuclear transport | 2.23E-04 | 0.088098764 |
| 130 | GO:0043486 | histone exchange | 2.32E-04 | 0.09014566 |
| 131 | GO:0090304 | nucleic acid metabolic process | 2.31E-04 | 0.09014566 |
| 132 | GO:1903827 | regulation of cellular protein localization | 2.36E-04 | 0.090789124 |
| 133 | GO:0006725 | cellular aromatic compound metabolic process | 2.38E-04 | 0.09104725 |
| 134 | GO:0016577 | histone demethylation | 2.46E-04 | 0.092600085 |
| 135 | GO:0012505 | endomembrane system | 2.45E-04 | 0.092600085 |
| 136 | GO:0006336 | DNA replication-independent nucleosome assembly | 2.63E-04 | 0.09788192 |
| 137 | GO:0034724 | DNA replication-independent nucleosome organization | 2.63E-04 | 0.09788192 |
| 138 | GO:0044260\|GO:0034960 | cellular macromolecule metabolic process | 2.65E-04 | 0.09790433 |
