# Supplementary material for: The miR-1224-5p/TNS4/EGFR axis inhibits tumour progression in oesophageal squamous cell carcinoma
Source: Cell Death Dis. 2020 Jul 30;11(7):597. doi: 10.1038/s41419-020-02801-6 (PMC7393493; doi:10.1038/s41419-020-02801-6)
Supplement: Supplementary file 5 — Table S5 [file 41419_2020_2801_MOESM5_ESM.docx]

**Table S5. Pathway analysis of differentially expressed genes in ESCC**

| NO. | Pathway | Number of entities | Matched with technology | Matched with entity list | *p* value |
| --- | --- | --- | --- | --- | --- |
| 1 | Alpha6Beta4Integrin | 53 | 49 | 4 | 0.02281995 |
| 2 | AndrogenReceptor | 98 | 93 | 6 | 0.01713533 |
