# Supplementary material for: The miR-1224-5p/TNS4/EGFR axis inhibits tumour progression in oesophageal squamous cell carcinoma
Source: Cell Death Dis. 2020 Jul 30;11(7):597. doi: 10.1038/s41419-020-02801-6 (PMC7393493; doi:10.1038/s41419-020-02801-6)
Supplement: Supplementary file 6 — Table S6 [file 41419_2020_2801_MOESM6_ESM.docx]

**Table S6. The correlation analysis of TNS4 and VEGFA protein expressions**

|  |  | TNS4 | | | |
| --- | --- | --- | --- | --- | --- |
|  |  | Positive | Negative | R | *p* value |
| VEGFA | Positive | 36 | 7 | 0.453 | 3.7718E-08 |
|  | Negative | 32 | 59 |  |  |
