# Supplementary material for: The miR-1224-5p/TNS4/EGFR axis inhibits tumour progression in oesophageal squamous cell carcinoma
Source: Cell Death Dis. 2020 Jul 30;11(7):597. doi: 10.1038/s41419-020-02801-6 (PMC7393493; doi:10.1038/s41419-020-02801-6)
Supplement: Supplementary file 7 — Table S7 [file 41419_2020_2801_MOESM7_ESM.docx]

**Table S7. The correlation between TNS4/VEGFA expressions and clinicopathological parameters**

| Parameter |  | TNS4 | | |  | VEGFA | | |  |
| --- | --- | --- | --- | --- | --- | --- | --- | --- | --- |
|  |  | Positive | Negative | *p* value |  | Positive | Negative | *p* value | |
| Gender |  |  |  | 0.6186 |  |  |  | 0.2158 | |
|  | Male | 52 | 48 |  |  | 35 | 65 |  | |
|  | Female | 16 | 18 |  |  | 8 | 26 |  | |
| Age |  |  |  | 0.8608 |  |  |  | 0.2393 | |
|  | ≤60 | 34 | 34 |  |  | 25 | 43 |  | |
|  | >60 | 34 | 32 |  |  | 18 | 48 |  | |
| pT |  |  |  | 0.6389 |  |  |  | 0.6213 | |
|  | T1-2 | 9 | 7 |  |  | 6 | 10 |  | |
|  | T3-4 | 59 | 59 |  |  | 37 | 81 |  | |
| pN |  |  |  | <0.0001 |  |  |  | 0.0161 | |
|  | N0 | 20 | 47 |  |  | 15 | 52 |  | |
|  | N1 | 48 | 19 |  |  | 28 | 39 |  | |
| Stage |  |  |  | 0.0019 |  |  |  | 0.728 | |
|  | 0-1 | 21 | 38 |  |  | 18 | 41 |  | |
|  | 2 | 47 | 28 |  |  | 25 | 50 |  | |
| Grade |  |  |  | 0.3785 |  |  |  | 0.4562 | |
|  | 1-2 | 48 | 51 |  |  | 30 | 69 |  | |
|  | 3 | 20 | 15 |  |  | 13 | 22 |  | |
