# Supplementary material for: The miR-1224-5p/TNS4/EGFR axis inhibits tumour progression in oesophageal squamous cell carcinoma
Source: Cell Death Dis. 2020 Jul 30;11(7):597. doi: 10.1038/s41419-020-02801-6 (PMC7393493; doi:10.1038/s41419-020-02801-6)
Supplement: Supplementary file 8 — Table S8 [file 41419_2020_2801_MOESM8_ESM.docx]

**Supplementary Figure Legends**

**Fig. S1 Low expression of miR-1224-5p is associated with asbestos exposure in non-small-cell lung cancer.** Transcript levels of miR-1224-5p in asbestos related NSCLC tissues and asbestos unrelated NSCLC tissues. NSCLC, non-small-cell lung cancer.

**Fig. S2 The expressions of PKD1, PPP1R9B, ACAP3 and HGS in esophageal cancer by analyzing TCGA dataset using GEPIA database.** * *p* < 0.05.

**Fig. S3 Knockdown of TNS4 inhibits the migration and invasion of KYSE150 and KYSE510 cells.** The migration and invasion abilities of TNS4 siRNAs transfected cells were measured by Transwell assay. Scale bar, 200 μm. Data are represented as the mean ± SD, and experiments were performed in triplicate. *** *p* < 0.001.

**Fig. S4 Knockdown of EGFR reduces VEGFA levels in the ESCC cell supernatants.** ELISA assay was used to examine the VEGFA levels in the ESCC cell supernatant. Data are represented as the mean ± SD, and experiments were performed in triplicate. ** *p* < 0.01, *** *p* < 0.001.
