# Supplementary figures and images for: The miR-1224-5p/TNS4/EGFR axis inhibits tumour progression in oesophageal squamous cell carcinoma
Source: Cell Death Dis. 2020 Jul 30;11(7):597. doi: 10.1038/s41419-020-02801-6 (PMC7393493; doi:10.1038/s41419-020-02801-6)

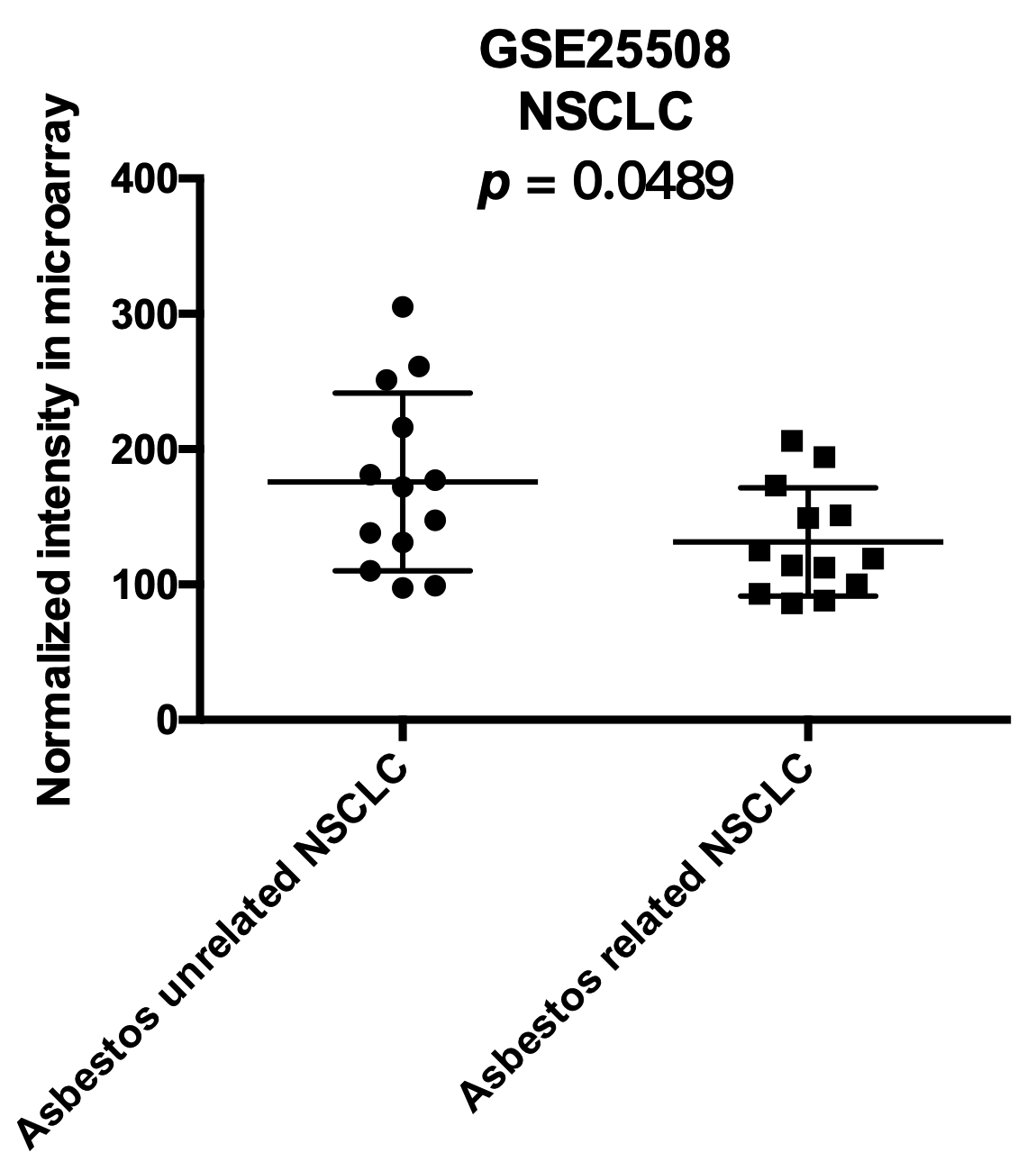

Supplement: Supplementary file 9 — Figure S1 [file 41419_2020_2801_MOESM9_ESM.tif]

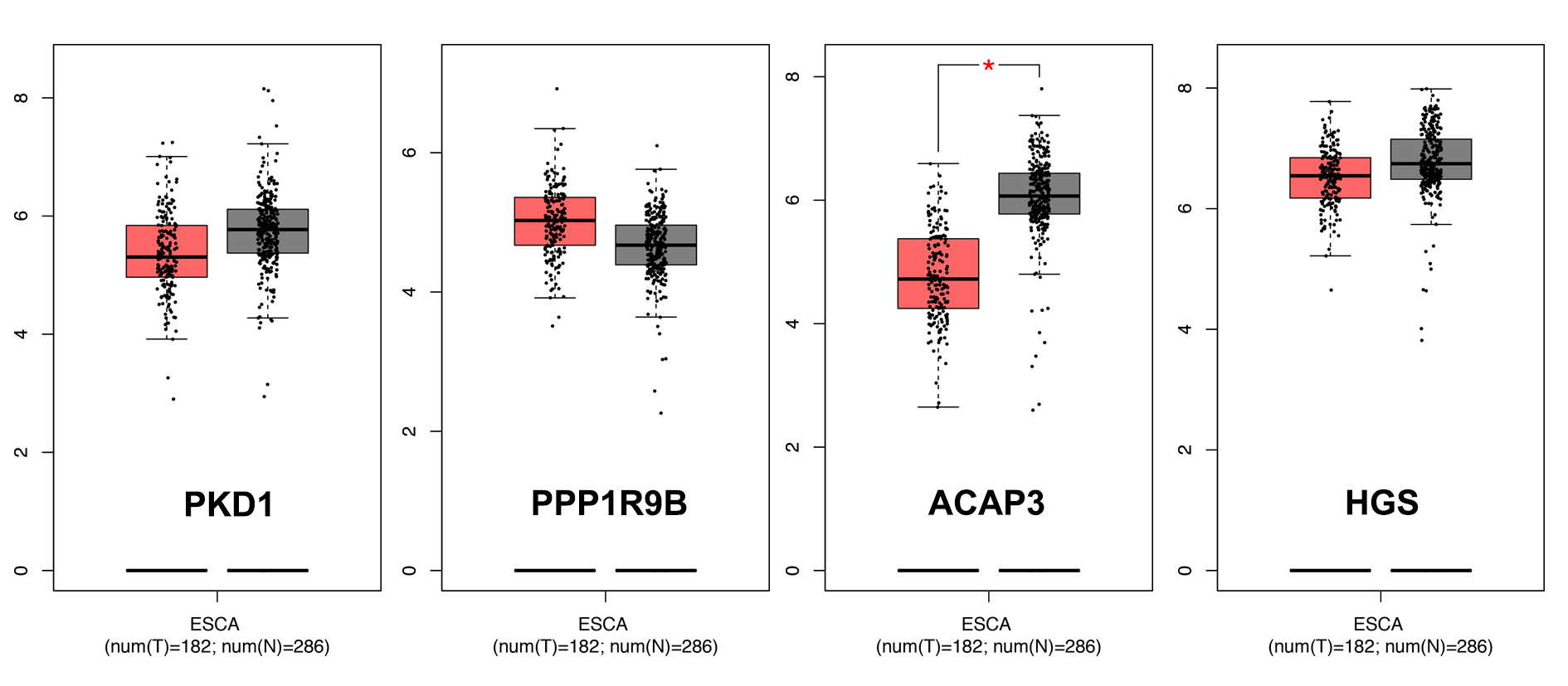

Supplement: Supplementary file 10 — Figure S2 [file 41419_2020_2801_MOESM10_ESM.tif]

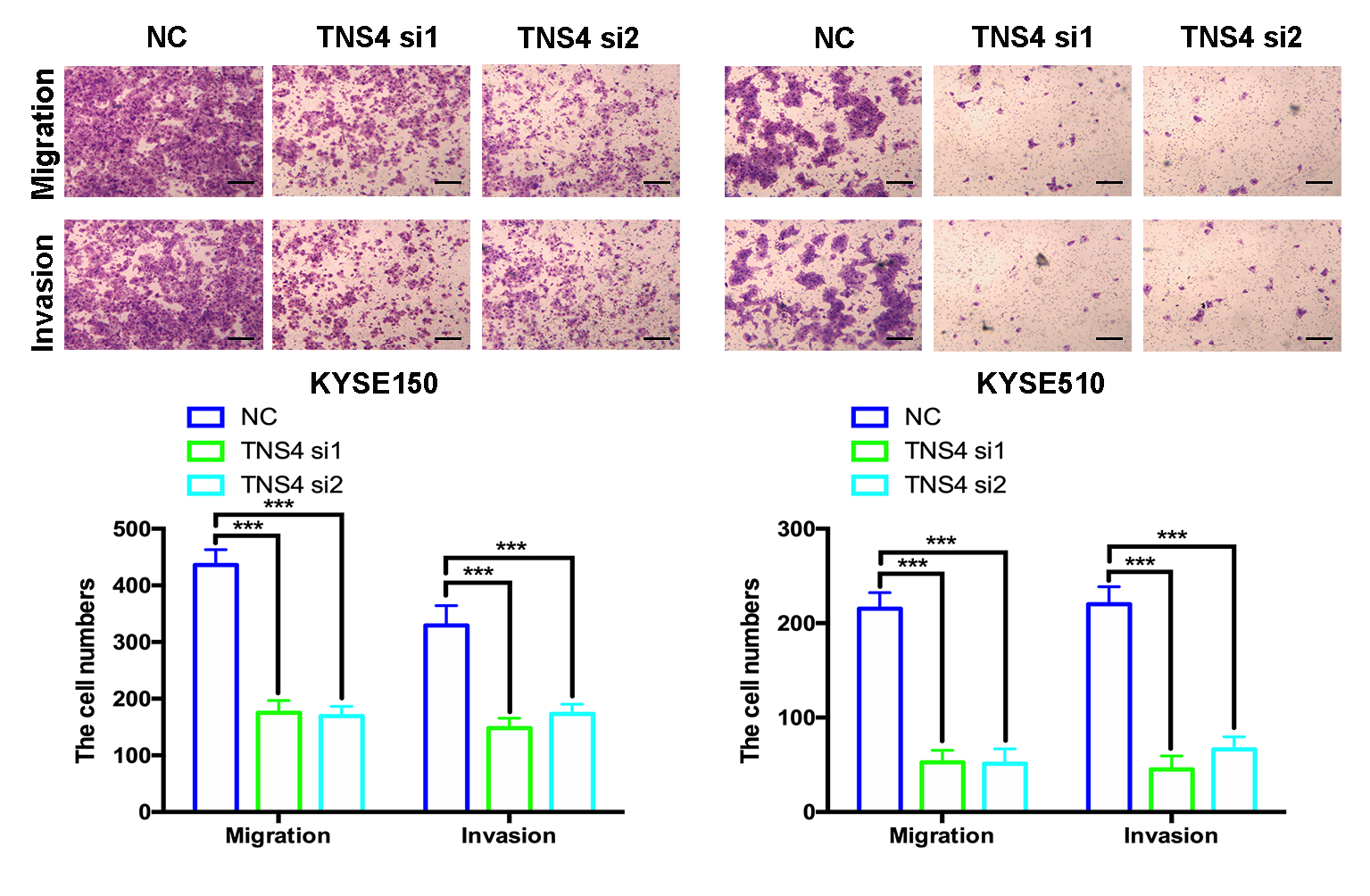

Supplement: Supplementary file 11 — Figure S3 [file 41419_2020_2801_MOESM11_ESM.tif]

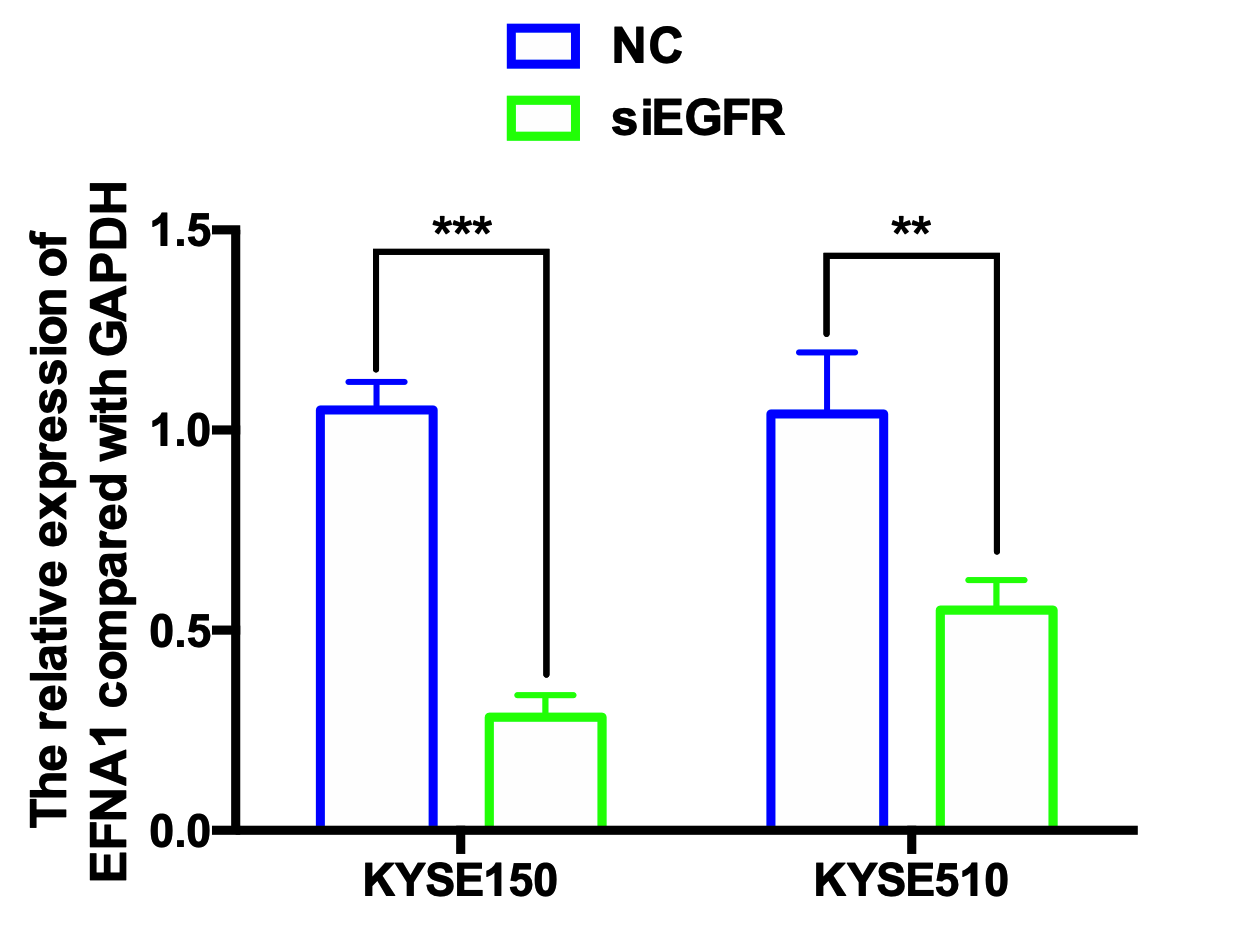

Supplement: Supplementary file 12 — Figure S4 [file 41419_2020_2801_MOESM12_ESM.tif]
